# Comparison of the effects of different traditional Chinese exercises on improving the motor function of stroke survivors: a network meta-analysis and systematic review

**DOI:** 10.3389/fneur.2026.1815489

**Published:** 2026-06-24

**Authors:** Runze Wang, Baicheng Ning, Shuangying Yang, Mingquan Li

**Affiliations:** 1 College of Integrated Chinese and Western Medicine, Changchun University of Chinese Medicine, Changchun, China; 2 School of Rehabilitation Medicine, Changchun University of Chinese Medicine, Changchun, China

**Keywords:** motor function, network meta-analysis, stroke, systematic review, traditional Chinese exercises

## Abstract

**Introduction:**

Stroke survivors frequently present with varying degrees of sensory, cognitive, language, and motor impairments. Traditional Chinese exercises have been widely used as adjunctive rehabilitation therapies because they combine low-to-moderate intensity physical training, balance practice, proprioceptive stimulation, motor relearning, respiratory regulation, and cognitive engagement. However, intervention protocols and outcome measures vary substantially across trials, and direct head-to-head comparisons among Tai Chi, Baduanjin, Wuqinxi, Yijinjing, and standard of care remain scarce. This network meta-analysis therefore compared the relative effectiveness of different traditional Chinese exercise modalities for improving post-stroke motor outcomes.

**Methods:**

We systematically searched Embase, Web of Science, Cochrane Library, PubMed, CNKI, VIP, and Wanfang Data from database inception to January 2026 for RCTs evaluating traditional Chinese exercises for post-stroke motor dysfunction. Eligible participants were adults with clinically diagnosed ischemic or hemorrhagic stroke in the acute, subacute, or chronic stage. The primary outcomes were upper- and lower-extremity motor function assessed using the FMA-UE and FMA-LE. Secondary outcomes were balance function assessed using the BBS and activities of daily living assessed using the BI. Risk of bias was assessed using the revised Cochrane risk-of-bias tool for randomized trials (RoB 2). A Bayesian network meta-analysis was performed, and intervention rankings were estimated using the SUCRA.

**Results:**

Fifty RCTs involving 3,718 participants were included. Compared with standard of care, Tai Chi and Wuqinxi showed statistically significant improvements in FMA-UE scores, whereas Baduanjin, Tai Chi, and Wuqinxi improved FMA-LE scores. All four exercise modalities were associated with significant improvements in BBS and BI scores. Ranking results suggested that Tai Chi had the highest probability of improving FMA-UE, Wuqinxi ranked highest for FMA-LE, Yijinjing ranked highest for BBS, and Baduanjin ranked highest for BI.

**Systematic review registration:**

https://www.crd.york.ac.uk/PROSPERO/view/CRD420261323853, CRD420261323853.

## 1 Introduction

Stroke is the second leading cause of death, and the third leading cause of disability among non-communicable diseases worldwide, and the number of individuals living with stroke-related disability has nearly doubled over the past three decades (1). Stroke survivors commonly experience varying degrees of motor, sensory, cognitive, and language impairments, and persistent functional deficits remain frequent even after transient ischemic attack or mild ischemic stroke (2). Motor impairment is the most prevalent post-stroke deficit, affecting up to 80% of survivors (3). Studies have suggested that post-stroke motor dysfunction is primarily attributable to impaired central motor signal transmission and compromised voluntary motor control. It may manifest as muscle paralysis or paresis, bradykinesia, and impaired coordination of the limbs and trunk. These symptoms may also contribute to the development of balance disorders (4, 5). The incidence of upper extremity weakness within the first 24 h after stroke onset is 35% (6). Subsequently, about half develop limb spasticity, with an incidence of 23–43% within the first six months after stroke, and moderate-to-severe cases accounting for up to 97% of those affected (7). Balance impairment occurs in approximately 83% of stroke survivors, substantially increasing fall risk and markedly reducing quality of life (8). Collectively, post-stroke motor dysfunction severely limits activities of daily living and imposes considerable caregiving and economic burdens on families and society, underscoring the urgent need for cost-effective therapeutic interventions.

Task-oriented training remains the cornerstone of rehabilitation for post-stroke motor dysfunction. Other therapeutic options include progressive therapeutic exercise, neurofunctional exercise-based rehabilitation, robot-assisted rehabilitation, rTMS, virtual-reality-based interventions, and neuromuscular electrical stimulation (9, 10). Previous studies suggest that these approaches can improve motor outcomes after stroke to some extent (3, 11); however, the certainty of the evidence and long-term effectiveness remain to be further investigated (9). In addition, technology-dependent interventions may increase financial burden, particularly in low- and middle-income settings.

In parallel, traditional Chinese exercise therapies (e.g., Tai Chi, Baduanjin, Wuqinxi, and Yijinjing) have been widely adopted as adjunctive interventions for post-stroke motor rehabilitation because they are generally low cost, accessible, adaptable, and compatible with supervised group or home-based practice. These exercises may hold clinical significance for stroke rehabilitation, as they integrate slow-paced movements, weight shifting, trunk rotation, coordinated upper and lower limb activities, proprioceptive stimulation, and balance training, particularly in the motor domain. Although several RCTs have reported beneficial effects of traditional Chinese exercises on post-stroke motor function, heterogeneity in intervention protocols and outcome measures, together with the lack of direct comparisons among different exercise modalities, limits conventional pairwise meta-analysis. Therefore, a network meta-analysis was necessary to integrate both direct and indirect evidence and to compare the relative efficacy of different traditional Chinese exercise modalities for limb motor performance, balance function, and activities of daily living.

## 2 Methods

We prepared the present study according to the Preferred Reporting Items for Systematic Reviews and Meta-Analyses (PRISMA) (Page et al., 2021). The study protocol has been registered in the International Prospective Register of Systematic Reviews (PROSPERO): https://www.crd.york.ac.uk/PROSPERO/home with the number of CRD420261323853.

### 2.1 Literature search

We conducted a systematic search of Embase, Web of Science, Cochrane Library, PubMed, CNKI, VIP, and Wanfang Data from database inception to January 2026. Search terms combined controlled vocabulary and free-text keywords related to stroke, motor dysfunction, and traditional Chinese exercise. No restriction was placed on publication language. Unpublished studies and gray literature were not included. We obtained the keywords from the MESH, the full search strategy is provided in Appendix a.

### 2.2 Inclusion and exclusion criteria

Eligibility criteria were defined according to the PICOS framework. Participants: adults with clinically diagnosed ischemic or hemorrhagic stroke and post-stroke motor dysfunction in the acute, subacute, or chronic stage; studies were analyzed together because several trials did not report stroke chronicity in a consistent manner. Interventions: traditional Chinese exercise interventions, including Tai Chi, Baduanjin, Wuqinxi, or Yijinjing, delivered alone or as an adjunct to standard rehabilitation. Comparators: SOC, conventional rehabilitation, usual care, or guideline-based rehabilitation provided by hospitals. Outcomes: the primary endpoints were limb motor function measured by FMA-UE and / or FMA-LE; secondary endpoints were BBS and BI. Higher scores on FMA-UE, FMA-LE, BBS, and BI indicate better upper-extremity motor function, lower-extremity motor function, balance performance, and activities of daily living, respectively. Study design: RCTs. Exclusion criteria were non-randomized studies, animal studies, letters, case reports, conference abstracts, reviews, unavailable full texts, duplicate publications, and studies without extractable data for the eligible outcomes. Intervention intensity, frequency, and duration were extracted, but subgroup analyses by these factors were not performed because reporting was inconsistent and the number of trials within each modality was limited.

### 2.3 Data extraction

Two reviewers initially screened the literature by examining titles and keywords to exclude studies that clearly did not meet the eligibility criteria. They then independently assessed the abstracts and full texts to determine final inclusion. Any disagreements were resolved through discussion with a third reviewer to reach a consensus. A standardized data extraction form was used to extract the following data: first author, publication year, country, study design, sample size, age, sex distribution, stroke stage when reported, intervention modality, intervention frequency, intervention duration, outcome measures, and adverse events.

### 2.4 Risk of bias assessment

Two reviewers independently used the Cochrane bias risk assessment tool version 2 (RoB 2) (12) to assess the risk of bias of RCTs, and negotiated with the third reviewer to resolve any discrepancies. Then, a schematic diagram of the bias risk assessment results was drawn by ReviewManager 5.3.

### 2.5 Outcome measures

Functional recovery was evaluated using the FMA, BBS, and BI, as they correspond to the key domains of post-stroke motor rehabilitation. FMA-UE consists of 33 items with a maximum score of 66, while FMA-LE comprises 17 items with a maximum score of 34; higher scores indicate better upper- or lower-extremity motor function. The BBS includes 14 items with a maximum score of 56, with higher scores indicating better balance performance and lower fall risk. The BI comprises 10 items with a maximum score of 100, with higher scores indicating greater independence in activities of daily living.

### 2.6 Data analysis

For any outcome addressed by two or more RCTs with similar interventions, we performed a meta-analysis to derive a pooled estimate. We used means ± standard deviations for continuous outcomes, and all indicators were represented using MD and 95% CI. We used R4.3.1 (R Foundation for Statistical Computing) and STATA15.0 (Stata Corporation, College Station, TX, USA) to perform a Bayesian network meta-analysis of the data to compare different interventions. The MCMC approach (13) was employed to generate pooled estimates and the corresponding probabilities for different treatment regimens, enabling the evaluation of their relative efficacy and ranking. The SUCRA was calculated to estimate the probability that each intervention would be the most effective for specific outcome measures related to post-stroke motor dysfunction.

Network plots and funnel plots were constructed using STATA 15.0 to illustrate both direct and indirect comparisons among treatment regimens and to assess potential publication bias and small-study effects. Cumulative ranking probability plots were generated using the ggplot2 package.

## 3 Results

### 3.1 Process and results of literature screening

**Figure 1:**
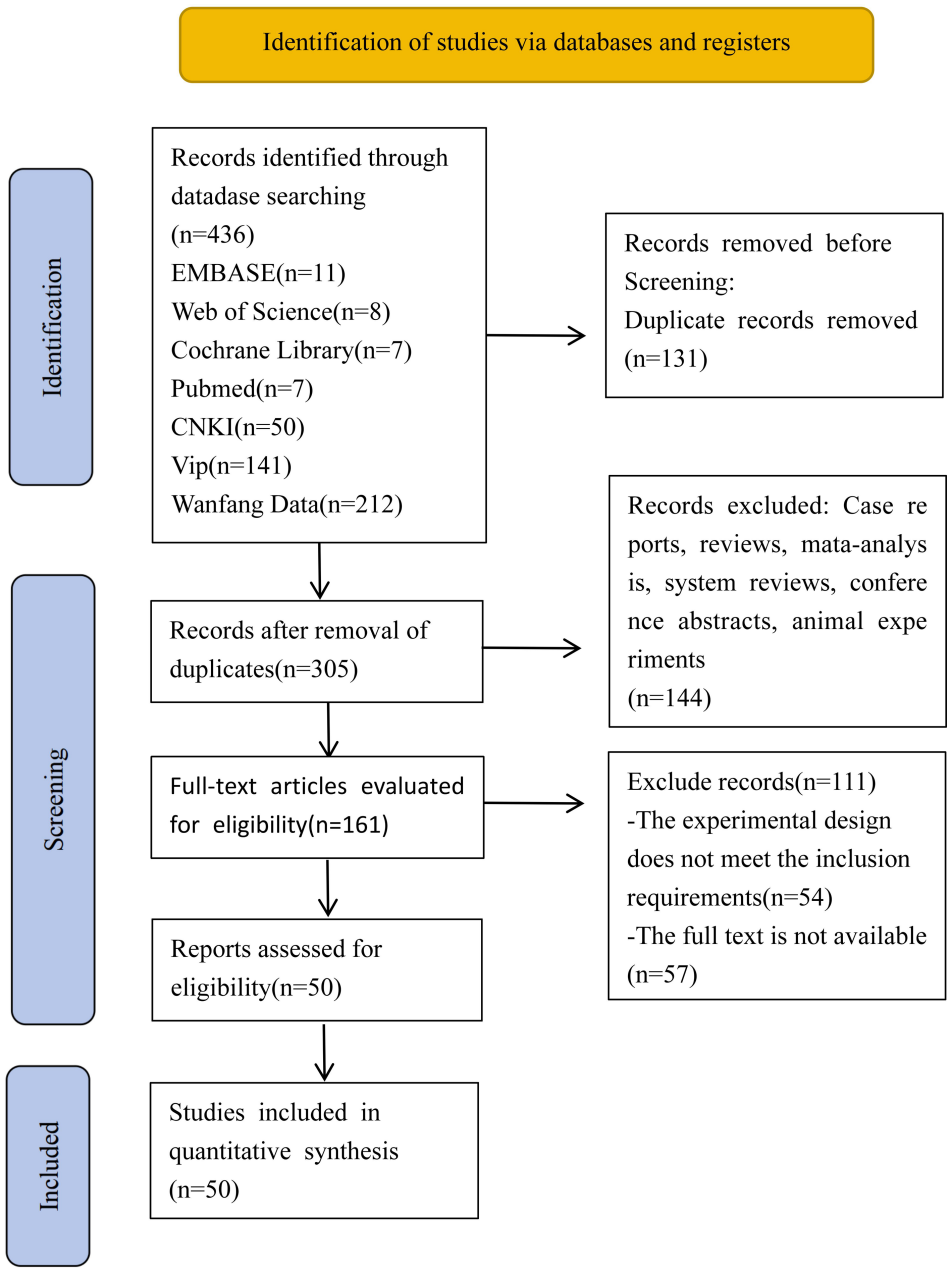
PRISlMA flow diagram of the study process.

We initially identified 436 records through database searching. After removing 131 duplicates, 305 records were screened by title and abstract. A total of 144 records, including reviews, systematic reviews, animal experiments, and clearly irrelevant studies, were excluded. After full-text assessment, 111 articles were excluded, and 50 RCTs were included (Figure 1).

### 3.2 Characteristics of included studies and demographic data

A total of 50 RCTs involving 3,718 participants were included in the Bayesian network meta-analysis, with an average sample size of approximately 74 participants per trial. Most trials were published in Chinese, and one trial was published in English. The average age of participants ranged from 45 to 80 years. Across the included studies, 25 reported FMA outcomes, 31 reported BBS outcomes, and 17 reported BI outcomes. The characteristics of the included studies are detailed in Table 1.

**Table 1:** Characteristics of included articles.

Included study	Country	Sample size	Age	Gender (M/F)	Experimental group	Basic features of experimental group	Outcome measures	Main conclusion
Ji, 2022 (35)	China	E: 45 C: 45	E: 69.32 ± 5.94 C: 68.42 ± 6.85	E: 26/19 C: 29/16	Ba Duan Jin	15 min/time, 6 times/week, 8 weeks	FMA	Baduanjin intervention improved motor function, enhanced lower-limb muscle activation patterns, and increased serum BDNF and NGF levels in hemiplegic stroke patients.
Ling, 2024 (36)	China	E: 49 C: 48	E: 53.32 ± 4.16 C: 51.45 ± 4.09	E: 28/21 C: 26/22	Ba Duan Jin	12 min/time, 5 times/week, 6 weeks	FMA BI	Baduanjin combined with early rehabilitation effectively improved motor function, daily living ability, disability status, and quality of life in patients with mild acute ischemic stroke.
Du, 2025 (37)	China	E: 70 C: 70	E: 62.87 ± 6.34 C: 61.23 ± 6.23	E: 48/22 C: 51/19	Ba Duan Jin	20 min/time, 2 times/day, 12 times/week, 4 weeks	BBS	Baduanjin training improved postural control, gait parameters, and balance function in convalescent stroke patients, supporting its value as a rehabilitation strategy.
Huang, 2025 (38)	China	E: 50 C: 50	E: 63.61 ± 6.43 C: 64.58 ± 6.73	E: 23/27 C: 26/24	Ba Duan Jin	30 min/time, 5 times/week, 6 weeks	FMA BBS BI	Baduanjin training significantly promoted limb functional recovery, enhanced daily living ability, and improved quality of life in patients during stroke recovery.
Xie, 2019 (39)	China	E: 20 C: 20	E: 51.10 ± 12.82 C: 53.95 ± 13.00	E: 13/7 C: 12/8	Ba Duan Jin	25 min/time, 5 times/week, 3 weeks	BBS BI	Baduanjin added to conventional rehabilitation improved motor function, balance, walking capacity, and activities of daily living more effectively than conventional rehabilitation alone.
Ding, 2019 (28)	China	E: 57 C: 56	E: 55.37 ± 4.71 C: 56.32 ± 3.17	E: 33/24 C: 31/25	Ba Duan Jin	20 min/time, 5 times/week, 4 weeks	BBS	Baduanjin Third Form combined with conventional balance training significantly improved balance function in stroke patients and was superior to conventional balance training alone.
Zhang, 2021 (40)	China	E: 41 C: 41	E: 71.28 ± 4.51 C: 70.45 ± 4.29	E: 23/18 C: 24/17	Ba Duan Jin	5 times/week 8 weeks	FMA BI	Baduanjin rehabilitation improved limb motor function, activities of daily living, and quality of life in elderly patients with post-stroke hemiplegia.
Liu, 2022 (41)	China	E: 50 C: 50	E: 55.32 ± 4.95 C: 55.41 ± 4.32	E: 26/24 C: 23/27	Ba Duan Jin	20-30 min/time, 3 times/week, 8 weeks	FMA	Baduanjin rehabilitation promoted neurological recovery, improved limb motor function, and enhanced static balance in patients during stroke recovery.
Zhou, 2021 (42)	China	E: 35 C: 35	E: 69.1 ± 8.5 C: 69.5 ± 8.3	E: 20/15 C: 22/13	Ba Duan Jin	60 min/time, 5 times/week, 12 weeks	BBS	Baduanjin combined with rehabilitation training reduced neurological impairment and improved balance, motor ability, and quality of life in elderly stroke patients.
Wang, 2023 (43)	China	E: 30 C: 30	E: 62.94 ± 6.17 C: 62.96 ± 6.18	E: 20/10 C: 21/9	Ba Duan Jin	20 min/time, 6 times/week, 4 weeks	BBS	Baduanjin exercise prescription improved balance ability, reduced anxiety, and showed favorable safety in convalescent stroke patients.
Guan, 2023 (44)	China	E: 50 C: 50	E: 70.07 ± 6.45 C: 70.19 ± 6.52	E: 29/21 C: 28/22	Ba Duan Jin	15 min/time, 12 weeks	FMA BBS BI	Baduanjin significantly improved neurological function, limb motor recovery, balance, and daily living ability in elderly convalescent stroke patients.
Chen, 2022 (45)	China	E: 46 C: 46	E: 53.12 ± 3.97 C: 52.48 ± 4.57	E: 31/15 C: 31/15	Tai Chi	30 min/time, 3 times/week 2 weeks	FMA BI	Tai Chi exercise showed beneficial effects in post-stroke hemiplegia rehabilitation by improving motor function, balance ability, and quality of life.
Zhao, 2025 (46)	China	E: 27 C: 27	E: 61.69 ± 8.61 C: 61.41 ± 8.92	E: 16/11 C: 15/12	Ba Duan Jin	40 min/time, 5 times/week, 6 weeks	FMA BBS	Six weeks of modified Baduanjin significantly improved lower-limb motor control, muscle coordination, and balance function in stroke patients.
Chen, 2024 (47)	China	E: 21 C: 21	E: 52.86 ± 14.84 C: 54.14 ± 12.80	E: 17/4 C: 17/4	Ba Duan Jin	40 min/time, 5 times/week, 4 weeks	FMA BBS BI	Modified Baduanjin exercise improved cardiopulmonary function and upper-limb motor function in stroke patients, suggesting clinical applicability as a safe rehabilitation exercise.
Fan, 2020 (48)	China	E: 43 C: 43	E: 64.3 ± 5.0 C: 63.8 ± 5.3	E: 29/14 C: 30/13	Tai Chi	90 min/time, 3 times/week, 12 weeks	FMA BBS	Modified Tai Chi improved balance, motor performance, and walking ability in stroke patients, potentially through downregulation of serum ALP, NPY, and IL-6 levels.
Zhou, 2015 (49)	China	E: 11 C: 11	Unclear	Unclear	Tai Chi	5 times/week 4 weeks	BBS	Modified Tai Chi significantly improved limb motor function and showed beneficial effects on balance recovery in patients with stroke.
Zhang, 2025 (22)	China	E: 68 C: 68	E: 62.04 ± 10.26 C: 64.67 ± 3.25	E: 41/27 C: 38/30	Wu Qin Xi	45 min/time, 12 weeks	FMA BBS BI	Modified Wuqinxi training reduced neurological deficits and improved motor function, balance, trunk control, and activities of daily living in elderly stroke patients.
Che, 2024 (23)	China	E: 28 C: 28	E: 64.23 ± 3.16 C: 65.07 ± 3.25	E: 22/6 C: 17/11	Wu Qin Xi	45 min/time, 5 times/week, 4 weeks	BBS	Modified Wuqinxi improved trunk control, postural stability, and balance in stroke patients; sacral-marker assessment, Pro-Kin balance testing, and BBS showed correlated evaluative value.
Zhang, 2023 (26)	China	E: 32 C: 28	E: 60.21 ± 9.02 C: 60.25 ± 9.03	E: 19/13 C: 15/13	Wu Qin Xi	30–50 min/time, 2 time/day 10 times/week, 12 weeks	BBS	Modified Wuqinxi improved immune-related indices, balance, gait parameters, and brain activation patterns in elderly stroke patients with gait and balance dysfunction.
Hou, 2025 (50)	China	E: 29 C: 27	E: 58.11 ± 11.32 C: 54.17 ± 11.94	E: 20/7 C: 23/6	Ba Duan Jin	30 min/time, 5 times/week, 8 weeks	BBS	Baduanjin improved core function, balance, and walking ability in stroke patients, with changes in core muscle EMG activity positively associated with balance improvement.
Cui, 2018 (51)	China	E: 24 C: 19	E: 53.67 ± 12.98 C: 55.33 ± 14.32	E: 15/9 C: 12/7	Ba Duan Jin	45 min/time, 5 times/week, 8 weeks	FMA	Baduanjin improved upper-limb motor function and balance function in patients recovering from stroke.
Zhang, 2010 (52)	China	E: 16 C: 16	Unclear	Unclear	Yi Jin Jing	45 min/time, 5 times/week, 2 weeks	FMA	Yijinjing exercise promoted upper- and lower-limb motor recovery in convalescent hemiplegic stroke patients and may serve as an effective therapeutic exercise method.
He, 2022 (53)	China	E: 29 C: 26	E: 62.96 ± 8.98 C: 62.50 ± 10.73	E: 23/6 C: 20/6	Tai Chi	40 min/time, 4 times/week, 4 weeks	FMA BBS	Tai Chi plus conventional rehabilitation improved selected postural balance parameters, especially backward and paretic-side control, more effectively than conventional rehabilitation alone.
Yang, 2013 (54)	China	E: 50 C: 50	E: 54.3 ± 13.8 C: 55.2 ± 14.6	E: 35/15 C: 31/19	Tai Chi	45 min/time, 6 times/week, 4 weeks	BBS BI	Tai Chi was an effective approach for post-stroke hemiplegic balance disorder, producing greater improvements in balance and activities of daily living than conventional rehabilitation.
Fu, 2016 (55)	China	E: 30 C: 30	E: 59.6 ± 7.6 C: 60.3 ± 8.4	E: 19/11 C: 18/12	Tai Chi	15 min/time, 6 times/week, 8 weeks	BBS	Tai Chi added to conventional rehabilitation significantly enhanced balance function and walking ability in convalescent stroke patients with hemiplegia.
Liu, 2019 (56)	China	E: 43 C: 44	Unclear	Unclear	Tai Chi	4 weeks	BBS	Tai Chi combined with routine rehabilitation effectively improved limb function, balance function, motor recovery, and quality of life in ischemic stroke patients.
Zhao, 2017 (57)	China	E: 30 C: 30	E: 53.85 ± 11.69 C: 51.38 ± 11.63	E: 20/10 C: 19/11	Tai Chi	30 min/time, 5 times/week, 8 weeks	BI	Tai Chi exercise improved depressive symptoms, motor function, and activities of daily living in patients with post-stroke depression, with greater benefits after sustained training.
Zhang, 2024 (58)	China	E: 35 C: 35	E: 64.71 ± 10.33 C: 68.47 ± 9.87	E: 20/15 C: 16/19	Tai Chi	30 min/time, 3 times/week, 4 weeks	FMA BBS	Tai Chi rehabilitation training improved postural balance, corrected abnormal posture, and supported functional recovery and quality-of-life improvement in stroke patients.
Wang, 2021 (18)	China	E: 30 C: 30	E: 73.3 ± 8.3 C: 73.6 ± 9.4	E: 22/8 C: 23/7	Tai Chi	20 min/time, 5 times/week, 40 days	BI	Tai Chi facilitated recovery of upper-limb function and improved activities of daily living in patients with post-stroke upper-limb dysfunction.
Jiang, 2018 (59)	China	E: 30 C: 30	E: 58.80 ± 11.70 C: 56.46 ± 12.81	E: 23/7 C: 22/8	Tai Chi	15 min/time, 2 times/day, 10times/week, 2 months	FMA	Tai Chi improved upper-limb proprioception, motor function, and coordination in hemiplegic stroke patients and may be suitable for community or home rehabilitation.
Xu, 2014 (60)	China	E: 40 C: 40	E: 60.14 ± 10.25 C: 48.23 ± 12.32	E: 22/18 C: 16/24	Tai Chi	20 min/time, 2 times/day, 12 weeks	BBS	Tai Chi promoted recovery of balance dysfunction in patients with post-stroke hemiplegia and improved lower-limb muscle strength.
Lai, 2024 (61)	China	E: 18 C: 18	E: 59.00 ± 9.17 C: 58.39 ± 9.42	E: 12/6 C: 11/7	Tai Chi	30 min/time, 3 times/week, 8 weeks	BBS	Tai Chi movements significantly improved walking function and balance in hemiplegic stroke patients.
Wang, 2023 (62)	China	E: 9 C: 8	E: 49.11 ± 11.85 C: 52.88 ± 11.79	E: 7/2 C: 6/2	Tai Chi	30 min/time, 7 times/week, 4 weeks	FMA BI	Tai Chi improved upper-limb motor function and shoulder flexion and abduction range of motion in Brunnstrom stage II stroke patients, supporting its role in early rehabilitation.
Xie, 2023 (63)	China	E: 42 C: 42	Unclear	Unclear	Tai Chi	5 times/week, 4 weeks	FMA	Compared with routine rehabilitation training, Tai Chi may improve the upper limb function of stroke patients by promoting routine muscle coordination and reducing compensatory movement patterns.
Yang, 2016 (64)	China	E: 28 C: 21	E: 51.43 ± 15.63 C: 54.85 ± 11.85	E: 17/11 C: 14/7	Tai Chi	40 min/time, 5 times/week, 2 months	FMA BBS	Tai Chi improved motor and balance function in stroke patients and may provide a feasible adjunctive strategy for hospital, community, and home-based rehabilitation.
Yang, 2019 (65)	China	E_1_: 28 E_2_: 23 C: 21	Unclear	E_1_: 17/11 E_2_: 14/9 C: 13/8	Tai Chi、 Ba Duan Jin	40 min/time, 5 times/week, 4 weeks	FMA	Tai Chi and Baduanjin enhanced agonist muscle strength in the hemiplegic lower limb, while Tai Chi appeared more effective in improving agonist–antagonist coordination.
Li, 2025 (66)	China	E: 10 C: 10	E: 56.00 ± 10.49 C: 62.00 ± 8.96	E: 5/5 C: 7/3	Ba Duan Jin	50 min/time, 5 times/week, 4 weeks	BBS	Tai Chi improved both static and dynamic balance in Brunnstrom stage IV stroke patients more effectively than conventional rehabilitation.
Zheng, 2020 (67)	China	E: 37 C: 37	E: 45.49 ± 11.15 C: 47.52 ± 13.83	E: 25/12 C: 27/10	Tai Chi	12 min/time, 12 weeks	BBS	Tai Chi combined with routine rehabilitation produced significant clinical benefits for post-stroke balance dysfunction and outperformed routine rehabilitation alone.
Zhu, 2025 (68)	China	E: 57 C: 57	E: 57.12 ± 5.74 C: 57.86 ± 5.81	E: 35/22 C: 37/20	Ba Duan Jin	20-30 min/time, 5 times/week, 4 weeks	BBS	Compared with core stability training, Baduanjin more effectively improved balance and reduced fear of movement or falling in convalescent stroke patients.
Liu, 2025 (69)	China	E: 49 C: 49	E: 73.33 ± 4.23 C: 72.68 ± 3.51	E: 21/28 C: 30/19	Ba Duan Jin	20-30 min/time, 2 times/day, 6 months	BBS	Baduanjin combined with exercise rehabilitation improved exercise tolerance, balance, neurological function, and quality of life in elderly hemiplegic stroke patients.
Luo, 2022 (70)	China	E: 24 C: 24	E: 68.08 ± 5.90 C: 66.25 ± 6.24	E: 19/5 C: 19/5	Yi Jin Jing	30 min/time, 5 times/week, 4 weeks	BBS	Yijinjing training improved paretic-limb weight-bearing capacity, center-of-gravity control, plantar pressure distribution, and balance function without reported adverse events.
Xu, 2022 (31)	China	E: 36 C: 38	E: 60.48 ± 7.89 C: 61.78 ± 8.06	E: 19/17 C: 22/16	Yi Jin Jing	15 min/time, 6 times/week, 2 weeks	BI	Yijinjing combined with conventional rehabilitation improved self-care ability, motor function, health status, balance, and negative emotions in severe hemiplegic stroke patients.
Xu, 2023 (25)	China	E: 30 C: 30	E: 64.29 ± 3.51 C: 63.51 ± 3.62	E: 18/12 C: 16/14	Yi Jin Jing	40 min/time, 1time/day, 4 times/week, 2 weeks	BBS BI	Yijinjing improved balance ability and fall efficacy in stroke patients, enhancing postural stability and confidence in performing daily activities without falling.
Tang, 2018 (71)	China	E: 30 C: 30	E: 59.2 ± 8.5 C: 59.5 ± 8.8	E: 15/15 C: 14/16	Wu Qin Xi	10 min/time, 5 times/week, 20 weeks	FMA	Modified Wuqinxi significantly improved motor function in middle-aged and elderly patients with post-stroke hemiplegia and was considered suitable for practice in this population.
Chen, 2024 (72)	China	E: 43 C: 43	E: 63.10 ± 4.11 C: 64.72 ± 3.65	E: 28/15 C: 27/16	Yi Jin Jing	40 min/time, 3 times/week, 4 weeks	FMA	Modified Yijinjing training improved upper limb motor function, including range of motion and coordination, in convalescent stroke patients.
Zhang, 2025 (73)	China	E: 69 C: 69	E: 60.56 ± 2.72 C: 60.38 ± 2.65	E: 39/30 C: 38/31	Yi Jin Jing	30 min/time, 1time/day, 5 times/week, 27 days	FMA BI	Modified Yijinjing therapy improved upper limb functional recovery and activities of daily living in stroke patients, demonstrating positive rehabilitation outcomes.
Wen, 2024 (74)	China	E: 41 C: 41	E: 62.53 ± 3.35 C: 62.59 ± 3.37	E: 24/17 C: 28/13	Wu Qin Xi	30 min/time, 5times/week, 8 weeks	BI	Wuqinxi training improved depressive symptoms, sleep quality, activities of daily living, and motor function in patients with post-stroke depression.
Chen, 2025 (75)	China	E: 61 C: 59	E: 66.85 ± 2.19 C: 66.19 ± 2.34	E: 28/33 C: 29/30	Tai Chi	45 min/time, 6 times/week, 4 weeks	FMA BI	Tai Chi promotes the recovery of upper limb function, improves joint mobility, enhances muscle activation, and improves daily living ability.
Zhang, 2025 (76)	China	E: 67 C: 56	E: 62.15 ± 2.91 C: 62.71 ± 3.03	E: 31/36 C: 29/27	Ba Duan Jin	60 min/time, 5 times/day, 6 months	FMA BBS	Baduanjin combined with conventional rehabilitation improved cardiopulmonary function, limb motor function, neurological function, balance, trunk control, gait parameters, and overall quality of life.
Chen, 2025 (24)	China	E: 30 C: 30	E: 60.70 ± 7.15 C: 59.33 ± 8.24	E: 21/9 C: 18/12	Wu Qin Xi	45 min/time, 5 times/day, 4 months	FMA BBS	Modified Wuqinxi combined with graded motor imagery improved limb function, balance, trunk coordination, and gait in patients with upper-limb hemiplegia after stroke.

C: Control; E: Experimental; M: Male; F: Female.

### 3.3 Risk of bias assessment

**Figure 2:**
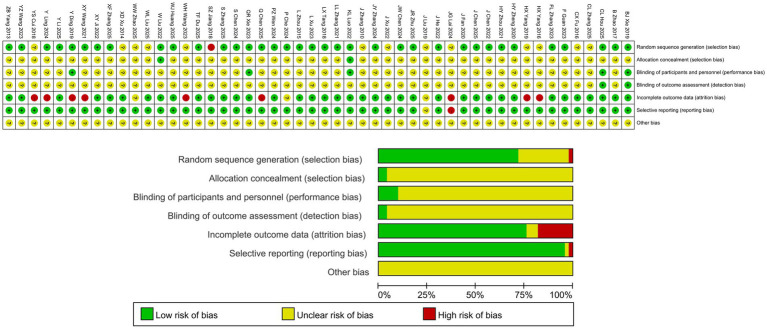
Risk of bias assessment of included studies.

The risk of bias assessment was conducted independently by two reviewers. Any disagreements were resolved through discussion with a third reviewer to achieve consensus. Of the included studies, five do clearly state blinding methods, and one study had a high risk of bias because it did not use random methods in the process of sequence generation, one had a high risk of bias because it did not report all pre-specified outcome indicators. The other ten high risks of bias arise mainly from incomplete data on outcome measures due to data loss. The risk of bias assessment of the included studies is shown in Figure 2.

## 4 Results of the network meta-analysis

### 4.1 FMA-UE score

**Figure 3:**
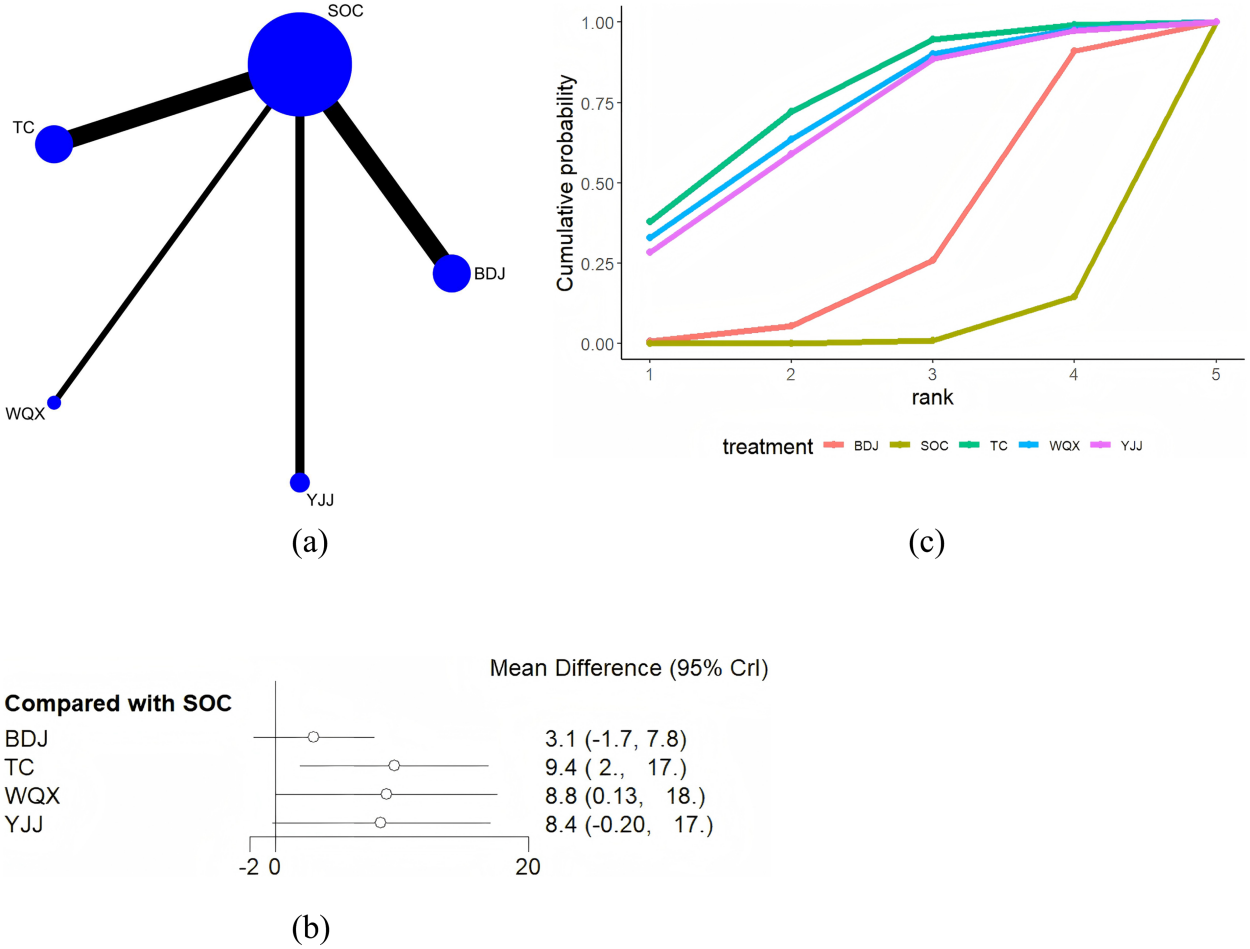
Effect of traditional Chinese exercises onFugl-Meyer Assessment for Upper Extremity effect.

As shown in Table 1 and Appendix b, a total of 19 studies reported FMA-UE outcomes. The network structure (Figure 3a) contained no closed loops, indicating that direct head-to-head comparisons among traditional exercise interventions were lacking and that the evidence was primarily derived from comparisons with SOC. Among the included studies, Baduanjin was the most frequently investigated intervention, followed by Tai Chi, whereas Wuqinxi and Yijinjing were less commonly evaluated (Table 2).

**Table 2:** Characteristics and clinical adaptation of traditional Chinese exercise modalities.

Exercise modality	Core training characteristics	Clinical adaptation for stroke survivors
Tai Chi	Slow continuous movements, weight shifting, trunk rotation, coordinated limb movement, attention control	Simplified forms, seated or supported standing practice, therapist guarding for high fall risk
Baduanjin	Eight structured routines, stretching, breathing regulation, trunk and limb coordination	Reduced movement amplitude and shorter sessions for patients with fatigue or spasticity
Wuqinxi	Imitation of five animal forms with stepping, squatting, reaching, and postural transitions	Modified forms and external support are needed for patients with severe weakness, spasticity, or poor independent standing
Yijinjing	Fascial stretching, isometric contraction, spiral or diagonal movement pattern.	Slow, low-load practice with close supervision when spasticity or compensatory movement is marked

The network meta-analysis results (Figure 3b) demonstrated that, compared with SOC, BDJ [MD = 3.1, 95% CI (−1.7, 7.8)] and YJJ [MD = 8.4, 95% CI (−0.20, 17.0)] did not show statistically significant effects on upper-extremity motor function. In contrast, TC [MD = 9.4, 95% CI (2.0, 17.0)] and WQX [MD = 8.8, 95% CI (0.13, 18.0)] were associated with significant improvements, with Tai Chi demonstrating the largest effect size. SUCRA rankings suggested that TC (76.0%) and WQX (71.0%) had the highest probabilities of effectiveness for FMA-UE, followed by YJJ (68.3%), BDJ (30.7%), and SOC (3.8%) (Figure 3c). Detailed estimates are provided in Appendix Table a1.

### 4.2 FMA-LE score

**Figure 4:**
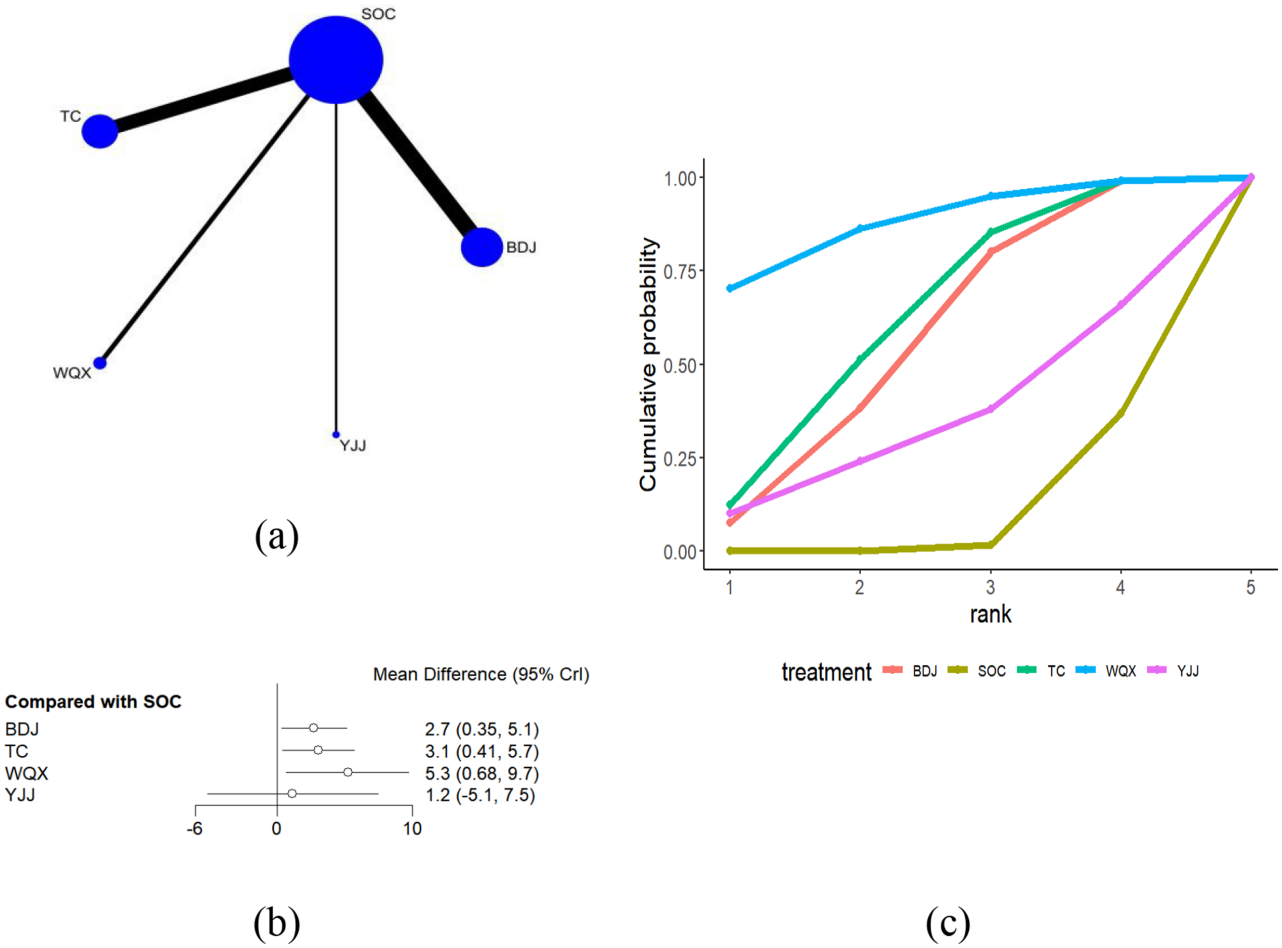
Effect of traditional Chinese exercises on Fugl-Meyer Assessment for Lower Extremity effect.

As shown in Table 1 and Appendix b, 16 studies reported FMA-LE outcomes. The network structure (Figure 4a) contained no closed loops, indicating a lack of direct head-to-head comparisons among traditional exercise interventions, with the evidence largely derived from comparisons with SOC. Among the included studies, Baduanjin was the most frequently investigated intervention, followed by Tai Chi, whereas Wuqinxi and Yijinjing were less commonly studied.

**Figure 5:**
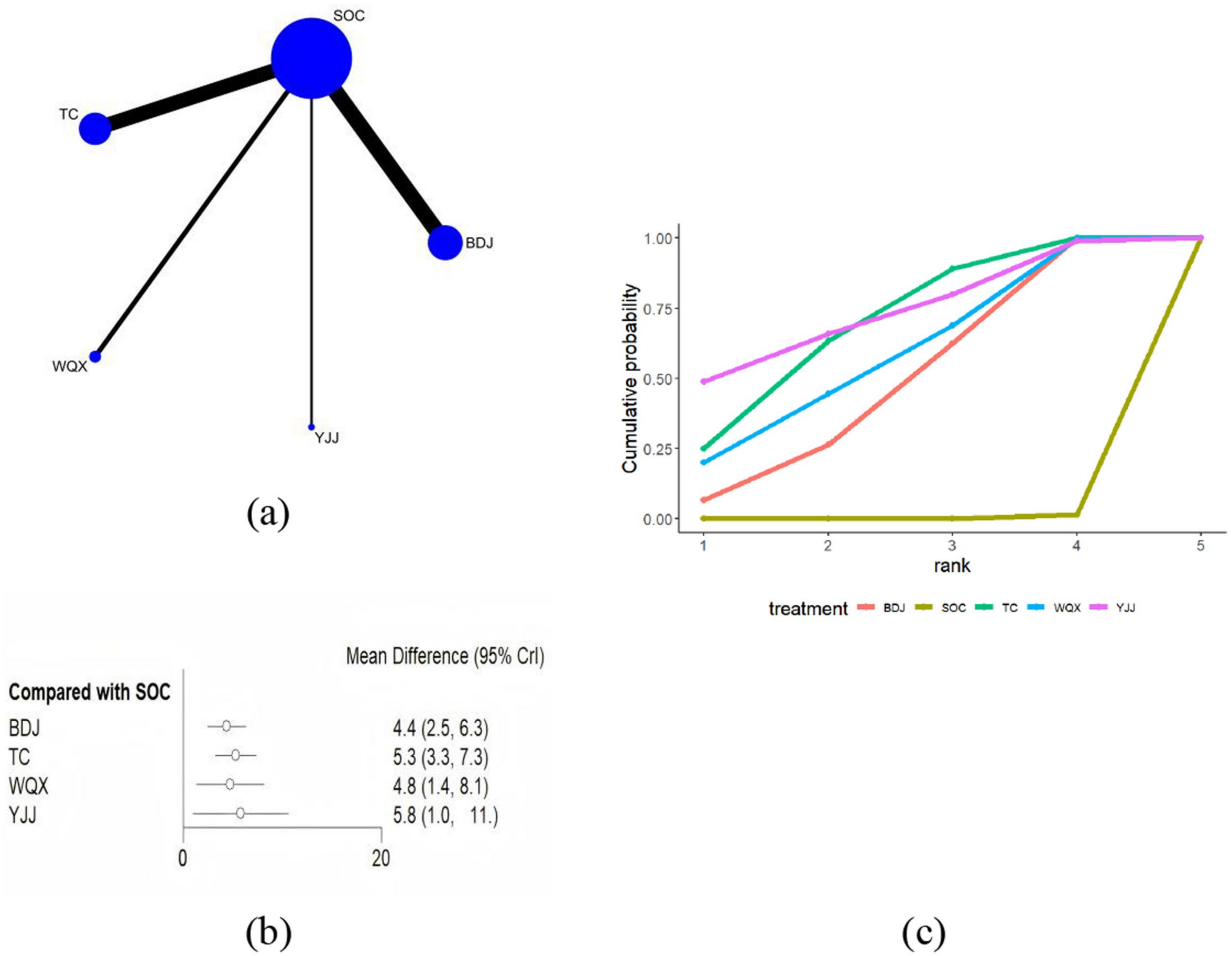
Effect of traditional Chinese exercises on Berg Balance scale effect.

The network meta-analysis results (Figure 4b) showed that, compared with SOC, BDJ [MD = 2.7, 95% CI (0.35, 5.1)], TC [MD = 3.1, 95% CI (0.41, 5.7)], and WQX [MD = 5.3, 95% CI (0.68, 5.7)] were associated with significant improvements in lower-extremity motor function, whereas YJJ [MD = 1.2, 95% CI(−5.1, 7.5)] did not reach statistical significance. Wuqinxi demonstrated the largest effect size and the highest SUCRA value for FMA-LE (87.7%), followed by TC (62.0%), BDJ (56.2%), YJJ (34.5%), and SOC (9.6%) (Figure 5c). Detailed estimates are presented in Appendix Table a2.

### 4.3 BBS score

As shown in Table 1 and Appendix b, a total of 31 studies reported BBS outcomes. The network structure (Figure 5a) contained no closed loops, indicating the absence of direct head-to-head comparisons among traditional exercise interventions, with the evidence primarily derived from comparisons with SOC. Among the included studies, Tai Chi and Baduanjin were the most frequently investigated interventions, followed by Yijinjing and Wuqinxi.

The network meta-analysis results (Figure 5b) demonstrated that, compared with SOC, BDJ [MD = 4.4, 95% CI (2.5, 6.3)], TC [MD = 5.3, 95% CI (3.3, 7.3)], WQX [MD = 4.8, 95% CI (1.4, 8.1)], and YJJ [MD = 5.8, 95% CI (1.0, 11.0)] were all associated with significant improvements in balance function among stroke survivors. Yijinjing showed the largest effect size and highest SUCRA value (73.4%), followed by TC (69.2%), WQX (58.2%), BDJ (48.8%), and SOC (0.3%). Because the number of Yijinjing trials was limited and the confidence interval was wide, this ranking should be interpreted cautiously. Detailed estimates are provided in Appendix Table a3.

### 4.4 BI score

**Figure 6:**
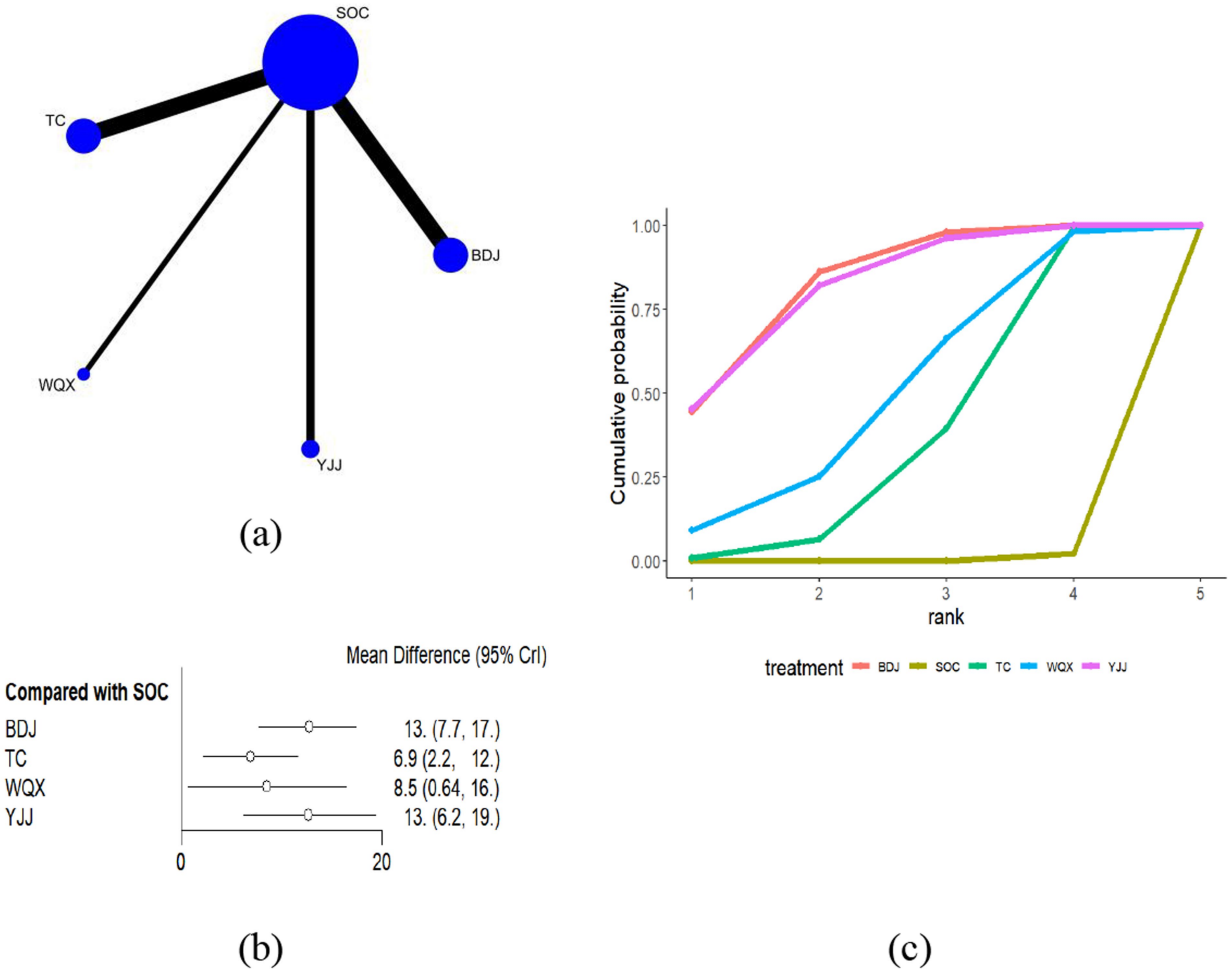
Effect of traditional Chinese exercises on Barthel Index effect.

As shown in Table 1 and Appendix b, 17 studies reported BI outcomes. The network structure (Figure 6a) contained no closed loops, indicating a lack of direct head-to-head comparisons among traditional exercise interventions, with the evidence primarily derived from comparisons with SOC. Among the included studies, Baduanjin and Tai Chi were the most frequently investigated interventions, followed by Yijinjing and Wuqinxi.

The network meta-analysis results (Figure 6b) demonstrated that, compared with SOC, BDJ [MD = 13.0, 95% CI (7.7, 17.0)], TC [MD = 6.9, 95% CI (2.2, 12.0)], WQX [MD = 8.5, 95% CI (0.64 to 16.0)], and YJJ [MD = 13.0, 95% CI (6.2 to 19.0)] were all associated with significant improvements in activities of daily living among stroke survivors. SUCRA rankings suggested that BDJ (82.1%) and WQX (80.9%) had the highest probabilities of improving BI, followed by YJJ (49.7%), TC (36.7%), and SOC (0.6%) (Figure 6c). Detailed estimates are presented in Appendix Table a4.

### 4.5 Pairwise comparisons

Across outcomes, Tai Chi and Wuqinxi were the most consistently associated with positive motor outcomes: Tai Chi ranked highest for FMA-UE and second for FMA-LE and BBS, whereas Wuqinxi ranked highest for FMA-LE and second for BI. The outcomes that improved most consistently were BBS and BI, for which all four traditional Chinese exercise modalities showed statistically significant advantages over SOC. Baduanjin and Tai Chi were the most frequently investigated interventions, suggesting greater clinical applicability and a stronger preliminary evidence base. No universally superior protocol emerged across all outcomes (Table 3).

**Table 3:** SUCRA of different Chinese medicine decoctions for various outcomes.

Treatment	BI (%)	BBS (%)	FMA LE (%)	FMA UE (%)
YJJ	80.9	73.4	34.5	68.3
WQX	49.7	58.2	87.7	71
BDJ	82.1	48.8	56.2	30.7
TC	36.7	69.2	62	76
SOC	0.6	0.3	9.6	3.8

### 4.6 Publication bias

We used scatter plots to assess publication bias among the included studies, with the results presented in Appendix Figures a1–a4. Different colors were applied to distinguish among the various traditional intervention modalities. The funnel plots for the FMA-UE and FMA-LE scores were generally symmetrical, indicating a low risk of publication bias. In contrast, the funnel plots for BBS and BI showed some asymmetry between the left and right sides, suggesting the presence of potential publication bias.

## 5 Discussion

Post-stroke motor dysfunction remains a major contributor to long-term disability. Research has indicated that high-quality rehabilitation should encompass multidisciplinary integration, early intervention, appropriately intense exercise, aerobic training, individualized programs, family involvement, and rehabilitation education. Such rehabilitation can effectively improve functional and emotional outcomes in stroke patients while reducing societal burden. Conversely, insufficient intensity or supervision, limited patient participation, or failure to consider the timing of rehabilitation initiation and multidisciplinary involvement may diminish the effectiveness of rehabilitation (14, 15). Traditional Chinese exercise modalities incorporate elements of low-to-moderate intensity training, individualized exercise prescriptions, and aerobic components. Therefore, under appropriate adaptation and supervision, these modalities may serve as complementary strategies for selected stroke survivors.

This study synthesized data from 50 RCTs to evaluate the effects of different traditional Chinese exercise modalities on post-stroke limb motor function, balance performance, and activities of daily living. The findings suggested outcome-specific differences rather than a single best exercise. Tai Chi showed the highest ranking probability for FMA-UE, Wuqinxi ranked highest for FMA-LE, Yijinjing ranked highest for BBS, and Baduanjin ranked highest for BI. However, because intervention protocols, exercise dose, supervision, outcome assessment, stroke severity, and stroke chronicity differed across studies, the rankings should be regarded as hypothesis-generating rather than definitive evidence of superiority.

The clinical presentation of post-stroke motor dysfunction exhibits significant heterogeneity, with its manifestation patterns depending on the lesion location, severity, and time since stroke onset. For instance, damage to the corticospinal tract may lead to spasticity and abnormal synergistic patterns, whereas injury to motor-related cortical areas may result in hemiplegia and muscle weakness (16). Balance dysfunction often coexists with these motor deficits; however, its underlying mechanisms are more complex, involving interactions across multiple systems, including sensory impairment, vestibular dysfunction, visual deficits, and fear of falling (17). Therefore, improvements in balance scores should not be interpreted as purely motor recovery. The outcome measures used in this review capture related but distinct dimensions: FMA-UE and FMA-LE mainly assess limb motor impairment, BBS assesses balance-related functional performance, and BI assesses independence in daily activities.

Furthermore, specific Tai Chi movements such as “Cloud Hands” involve bilateral alternating coordination patterns that resemble some symmetrical and asymmetrical movement strategies emphasized in PNF. However, unlike therapist-applied PNF, Tai Chi usually relies on active or active-assisted movement with verbal instruction, visual demonstration, guarding, and environmental support. For patients with severe weakness, poor balance, or spasticity, practice should begin with seated or supported standing versions, smaller movement amplitudes, slower transitions, and therapist assistance to prevent falls. These movements incorporate shoulder abduction, wrist extension, and forearm pronation–supination, potentially facilitating proprioceptive reorganization and upper-extremity motor recovery (18). Previous studies have reported that Tai Chi significantly improves BBS scores as well as standing and walking performance in stroke survivors (19), with targeted movements contributing to upper-limb functional gains (20). Long-term practice has also been associated with improved hand–eye coordination, enhanced daily functioning, and reduced fall risk in older adults (21).

For FMA-LE outcomes, Wuqinxi demonstrated the largest treatment effect, followed by Tai Chi and Baduanjin. The FMA-LE subscale evaluates multiple domains of lower-extremity motor function, including reflex activity, movement coordination and balance-related tasks. Because original Wuqinxi routines can be complex and physically demanding, most therapists use simplified or modified forms (22–24). For example, bear-form semi-squat and weight-shifting tasks may increase controlled loading of the affected limb and strengthen hip and knee control, whereas deer-form trunk rotation may facilitate turning stability. Monkey-form reaching and stepping may support anticipatory postural adjustment, and bird-form single-leg or supported stance tasks may challenge ankle control and static balance (24).

Collectively, the multidirectional loading, stepping, and postural transitions characteristic of modified Wuqinxi may promote lower-extremity recovery through repeated task-specific practice, progressive loading of the paretic limb, enhanced proprioceptive feedback, and reduction of compensatory movement strategies. These mechanisms provide a plausible explanation for their relatively greater impact on the FMA-LE; however, the exact mechanisms still require further clinical validation.

Multiple clinical studies have demonstrated that Wuqinxi training can improve lower-extremity joint mobility, enhance muscle strength, and increase balance performance in stroke survivors (22, 25). In addition, sustained Wuqinxi practice has been associated with reductions in neurological deficit severity following stroke (23).

Zhang et al. (26) evaluated stroke patients after three months of Wuqinxi training using functional magnetic resonance imaging, cerebral hemodynamic assessment, and serum immunological markers. The intervention group showed activation in brain regions including the occipital cortex, basal ganglia, and thalamus, alongside improvements in mean cerebral arterial flow velocity, vascular resistance index, and cerebral blood flow volume compared with controls. Serum immunoglobulin levels (IgG, IgA, and IgM) were also increased. These findings suggest that, as a low-to-moderate intensity aerobic exercise, long-term Wuqinxi practice may facilitate recovery through improved cerebral perfusion, enhanced neuroplasticity, and increased immune activity. Furthermore, a study involving healthy university students reported that 15 weeks of Wuqinxi training significantly improved cardiopulmonary fitness, flexibility, muscle strength, and endurance, providing additional physiological evidence supporting the effectiveness of this modality (27). Collectively, these findings indicate that Wuqinxi may promote post-stroke lower-extremity recovery through combined mechanisms involving motor control reorganization, cerebral hemodynamic enhancement, and systemic physiological adaptation.

The BI is a widely used measure of ADL in individuals with functional impairment. In the present analysis, Baduanjin demonstrated the largest effect size for BI improvement, followed by Yijinjing, although the difference between the two interventions was not statistically significant. Baduanjin consists of eight structured movements characterized by slow tempo, moderate intensity, and high feasibility, which may support patient adherence. The practice primarily targets trunk and lower-limb core musculature while emphasizing coordinated control between the trunk and extremities. Through multidirectional muscle stretching, joint mobilization, breathing regulation, and fine motor tasks such as grasping, Baduanjin may improve sensory input, muscle tone, and motor control on the affected side, thereby enhancing joint mobility (28). Previous meta-analyses have reported that Baduanjin significantly improves multiple functional outcomes, including ADL performance (29). Short-term training has also been shown to increase the thickness of the transversus abdominis and multifidus, contributing to improved core stability and trunk control (30).

Yijinjing places greater emphasis on deep fascial stretching and isometric contraction, making it particularly suitable for patients with spasticity and pronounced compensatory movement patterns. Its training principles share similarities with core stability training, which may facilitate improvements in strength and balance (31). A study involving 74 patients with severe post-stroke hemiplegia reported that adding Yijinjing to conventional rehabilitation for 3 months significantly improved quality of life, overall health status, and negative emotional outcomes compared with controls (31). In addition, Yijinjing has been associated with improvements in cardiopulmonary function, modulation of heart rate variability, optimization of breathing rhythm, and enhanced fatigue tolerance, thereby supporting exercise sustainability and efficiency (32). Overall, Baduanjin and Yijinjing may enhance ADL performance through combined mechanisms including muscle and fascial stretching, joint mobility training, fine motor practice, improved coordination, and enhanced soft-tissue circulation.

This study explored the differences in efficacy among various traditional Chinese exercises in improving post-stroke motor-related outcomes. Compared with SOC alone, traditional Chinese exercises appeared to provide additional benefits for limb motor function, balance performance, and activities of daily living. Some studies have suggested that higher doses and greater intensities of exercise are more beneficial for improving motor function. However, for stroke patients—particularly those in the early stages—prolonged exercise may induce fatigue, which could compromise the quality of rehabilitation. Therefore, standardized assessment of fatigue should be considered for stroke patients (33). Furthermore, rehabilitation outcomes are influenced not only by exercise modality but also by stroke severity, lesion location, baseline disability, cognition, depression, frailty, nutrition, pain, fatigue, spasticity severity, and adherence. Medical complications during rehabilitation hospitalization may further delay recovery. For example, infections such as *Clostridium difficile* can reduce participation in rehabilitation, prolong hospitalization, and negatively influence functional outcomes (34). Therefore, the efficacy of exercise interventions should be interpreted within the broader context of multidisciplinary medical management and patient-specific risk factors.

A major strength of this study lies in the application of a Bayesian network meta-analysis to compare the effects of different traditional Chinese exercises on functional outcomes in stroke survivors. By integrating direct and indirect evidence across specific outcome measures, this approach provided new insights for optimizing rehabilitation strategies. However, several limitations should be acknowledged. Some included studies had small sample sizes and variable methodological quality; substantial heterogeneity existed in intervention duration and frequency; and due to the limited number of available studies, other forms of traditional exercises were not included. Furthermore, most trials compared a single exercise modality with SOC rather than with another active, conventional exercise approach. This results in the absence of a closed-loop network, thereby limiting the certainty of comparisons between different exercise modalities. Finally, most outcomes were assessed using clinical rating scales, with limited application of objective neurophysiological or biomechanical measures.

## 6 Conclusion

Traditional Chinese exercises appear promising as adjunctive rehabilitation approaches for improving motor-related outcomes after stroke. The current evidence suggests that Tai Chi may be more favorable for upper-extremity motor function, Wuqinxi for lower-extremity motor function, Yijinjing for balance performance, and Baduanjin for activities of daily living. However, no universally superior exercise protocol can be concluded because the included trials differed substantially in participant characteristics, stroke stage, intervention dose, supervision, and outcome measures, and most comparisons were indirect. Exercise selection should therefore be individualized according to rehabilitation goals, stroke severity, balance ability, spasticity, safety risk, patient preference, and available therapist support. Further well-designed RCTs with standardized protocols and comprehensive safety reporting are needed to confirm these findings. Future research should conduct large-scale, high-quality RCTs with standardized protocols, clear therapist training requirements, prospective adverse-event monitoring, subgroup analyses by stroke stage and severity, and multidimensional assessments including fatigue, adherence, gait, and neurophysiological indicators.

## Data Availability

The datasets presented in this study can be found in online repositories. The names of the repository/repositories and accession number(s) can be found in the article/Supplementary material.

## Glossary

CNKI: China National Knowledge Infrastructure
VIP: Chinese Scientific Journal Database
MESH: Medical Subject Headings
rTMS: repetitive Transcranial Magnetic Stimulation
BDJ: Baduanjin
TC: Tai Chi
WQX: Wuqinxi
YJJ: Yijinjing
FMA-UE: Fugl-Meyer Assessment for Upper Extremity
FMA-LE: Fugl-Meyer Assessment for Lower Extremity
RCTs: Randomized Controlled Trials
BI: Barthel Index
BBS: Berg Balance scale
SOC: Standards of Care
MCMC: Markov Chain Monte Carlo
SUCRA: Surface Under the Cumulative Ranking Curve
MD: mean difference
95% CI: 95% confidence interval
PNF: proprioceptive neuromuscular facilitation
IgG: lmmunoglobin G
IgA: lmmunoglobin A
IgM: lmmunoglobin M
ADL: Activity of Daily Living
