# Supplementary material for: Comparison of the effects of different traditional Chinese exercises on improving the motor function of stroke survivors: a network meta-analysis and systematic review
Source: Front Neurol. 2026 Jun 24;17:1815489. doi: 10.3389/fneur.2026.1815489 (PMC13341441; doi:10.3389/fneur.2026.1815489)
Supplement: Supplementary file 1 [file Supplementary_file_1.PDF]

Appendix a: Common database retrieval strategies

Database	Retrieval strategy
EMBASE	#1 'accident, cerebrovascular':ti,ab,kw OR 'acute cerebrovascular lesion':ti,ab,kw OR 'acute focal cerebral vasculopathy':ti,ab,kw OR 'acute stroke':ti,ab,kw OR 'apoplectic stroke':ti,ab,kw OR apoplexia:ti,ab,kw OR apoplexy:ti,ab,kw OR 'blood flow disturbance, brain':ti,ab,kw OR 'brain accident':ti,ab,kw OR 'brain attack':ti,ab,kw OR 'brain blood flow disturbance':ti,ab,kw OR 'brain insult':ti,ab,kw OR 'brain insultus':ti,ab,kw OR 'brain vascular accident':ti,ab,kw OR 'cerebral apoplexia':ti,ab,kw OR 'cerebral insult':ti,ab,kw OR 'cerebral stroke':ti,ab,kw OR 'cerebral vascular accident':ti,ab,kw OR 'cerebral vascular insufficiency':ti,ab,kw OR 'cerebro vascular accident':ti,ab,kw OR 'cerebrovascular arrest':ti,ab,kw OR 'cerebrovascular failure':ti,ab,kw OR 'cerebrovascular injury':ti,ab,kw OR 'cerebrovascular insufficiency':ti,ab,kw OR 'cerebrovascular insult':ti,ab,kw OR 'cerebrum vascular accident':ti,ab,kw OR 'cryptogenic stroke':ti,ab,kw OR cva:ti,ab,kw OR 'nsultus cerebralis':ti,ab,kw OR 'ischaemic seizure':ti,ab,kw OR 'ischemic seizure':ti,ab,kw OR stroke:ti,ab,kw OR 'thrombotic stroke':ti,ab,kw OR 'cerebrovascular accident':ti,ab,kw<br>#2 'disability, motor':ti,ab,kw OR 'motor disability':ti,ab,kw OR 'motor disfunction':ti,ab,kw OR 'motor disorder':ti,ab,kw OR 'motor disorders':ti,ab,kw OR 'motor disturbance':ti,ab,kw OR 'motor impairment':ti,ab,kw OR 'movement disorder':ti,ab,kw OR 'movement disorders':ti,ab,kw OR 'motor dysfunction':ti,ab,kw OR 'balancing function':ti,ab,kw<br>#3 'tai chi':ti,ab,kw OR 'qi gong':ti,ab,kw OR 'ba duan jin':ti,ab,kw OR 'wu qin xi':ti,ab,kw OR 'yi jin jing':ti,ab,kw<br>#1 AND #2 AND #3
Web of Science	#1 ((TS=(motor dysfunction)) OR TS=(dysfunction)) OR TS=(Balancing function)<br>#2 (((((((((((((((TS=(Strokes)) OR TS=(Strokes)) OR TS=(Cerebrovascular Accident)) OR TS=(Cerebral Stroke)) OR TS=(Stroke, Cerebral)) OR TS=(Cerebrovascular Apoplexy)) OR TS=(Apoplexy, Cerebrovascular)) OR TS=(Vascular Accident)) OR TS=(Brain Vascular Accident)) OR TS=(Vascular Accidents, Brain)) OR TS=(Stroke, Cerebrovascular)) OR TS=(Cerebrovascular Stroke)) OR TS=(Apoplexy)) OR TS=(Stroke, Acute)) OR TS=(Cerebrovascular Accident, Acute)) OR TS=(Cerebrovascular Accident)) OR TS=(CVA)<br>#3 ((((TS=(tai chi)) OR TS=(Qi gong)) OR TS=(Ba Duan Jin)) OR TS=(Wu Qin Xi)) OR TS=(Yi Jin Jing)<br>#4 #1 AND #2 AND #3
Cochrane Library	#1 MeSH descriptor: [Stroke] explode all trees<br>#2 (Strokes):ti,ab,kw OR (Strokes OR CVA (Cerebrovascular Accident) OR Apoplexy OR Cerebrovascular Accident OR Cerebrovascular Stroke OR Vascular Accident, Brain OR Stroke, Cerebrovascular OR Cerebral Strokes OR Vascular Accidents, Brain OR Strokes, Cerebral OR Cerebrovascular Accidents OR Strokes, Cerebrovascular OR Brain Vascular Accident OR Cerebral Stroke OR Cerebrovascular Apoplexy OR Stroke, Cerebral OR Apoplexy OR Cerebrovascular OR CVAs (Cerebrovascular Accident) OR Cerebrovascular Accidents, Acute OR Acute Cerebrovascular Accident Stroke, Acute OR Strokes, Acute):ti,ab,kw<br>#3 (Tai Chi):ti,ab,kw OR (Ba Duan Jin):ti,ab,kw OR (Yi Jin Jing):ti,ab,kw OR (Wu Qin XI):ti,ab,kw OR (QI Gong):ti,ab,kw<br>#4 (Balancing function OR dysfunction OR motor dysfunction OR disability, motor OR motor disability OR motor disfunction OR motor disorder OR motor disorders OR motor disturbance OR motor impairment OR movement disorder OR movement disorders OR motor dysfunction):ti,ab,kw<br>#5 #1 AND #2 AND #3 AND #4
Pubmed	#1 (((((tai chi[Title/Abstract])) OR (Ba Duan Jin[Title/Abstract])) OR (Wu Qin Xi[Title/Abstract])) OR (Yi Jin Jing[Title/Abstract])) OR (Qi gong[Title/Abstract])<br>#2 ((((((((((((((((((((((((((((("Stroke"[Mesh]) OR (Strokes[Title/Abstract])) OR (Cerebrovascular Accident[Title/Abstract])) OR (Cerebrovascular Accidents[Title/Abstract])) OR (Cerebral Stroke[Title/Abstract])) OR (Cerebral Strokes[Title/Abstract])) OR (Stroke, Cerebral[Title/Abstract])) OR (Strokes, Cerebral[Title/Abstract])) OR (Cerebrovascular Apoplexy[Title/Abstract])) OR (Apoplexy, Cerebrovascular[Title/Abstract])) OR (Vascular Accident, Brain[Title/Abstract])) OR (Brain Vascular Accident[Title/Abstract])) OR (Brain Vascular Accidents[Title/Abstract])) OR (Vascular Accidents, Brain[Title/Abstract])) OR (Cerebrovascular Stroke[Title/Abstract])) OR (Cerebrovascular Strokes[Title/Abstract])) OR (Stroke, Cerebrovascular[Title/Abstract])) OR (Strokes, Cerebrovascular[Title/Abstract])) OR (Apoplexy[Title/Abstract])) OR (CVA (Cerebrovascular Accident[Title/Abstract]))) OR (CVAs (Cerebrovascular Accident[Title/Abstract]))) OR (Stroke, Acute[Title/Abstract])) OR (Acute Stroke[Title/Abstract])) OR (Acute Strokes[Title/Abstract])) OR (Strokes, Acute[Title/Abstract])) OR (Cerebrovascular Accident, Acute[Title/Abstract])) OR (Acute Cerebrovascular Accident[Title/Abstract])) OR (Acute Cerebrovascular Accidents[Title/Abstract])) OR (Cerebrovascular Accidents, Acute[Title/Abstract])))<br>#3 ((((((((((motor dysfunction[Title/Abstract]) OR (disability, motor[Title/Abstract])) OR (motor disability[Title/Abstract])) OR (motor disfunction[Title/Abstract])) OR (motor disorder[Title/Abstract])) OR (motor disorders[Title/Abstract])) OR (motor disturbance[Title/Abstract])) OR (motor impairment[Title/Abstract])) OR (movement disorder[Title/Abstract])) OR (movement disorders[Title/Abstract])) OR (motor dysfunction[Title/Abstract])<br>#4 #1 and #2 and #3
CNKI	（主题：卒中后运动功能障碍 + 脑卒中后运动功能障碍 + 中风后运动功能障碍 + 生活质量 + 平衡功能 + 功能障碍 + 运动功能 + 卒中 + 中风）AND（主题：太极 + 太极拳 + 八段锦 + 五禽戏 +易筋经 + 气功 + 健身气功）AND（篇关摘：FMA +BBS + BI(模糊)）
VIP	((((((((((题名或关键词=卒中后运动功能障碍 OR 题名或关键词=脑卒中后运动功能障碍) OR 题名或关键词=中风后运动功能障碍) OR 题名或关键词=生活质量) OR 题名或关键词=平衡功能) OR 题名或关键词=功能障碍) OR 题名或关键词=运动功能) OR 题名或关键词=卒中) OR 题名或关键词=脑卒中) OR 题名或关键词=中风) AND ((((((题名或关键词=太极 OR 题名或关键词=太极拳) OR 题名或关键词=八段锦) OR 题名或关键词=五禽戏) OR 题名或关键词=易筋经) OR 题名或关键词=气功) OR 题名或关键词=健身气功)) AND ((摘要=FMA OR 摘要=BBS) OR 摘要=BI))
Wanfang Data	检索表达式（中英文扩展）： 主题:(卒中后运动功能障碍 OR 脑卒中后运动功能障碍 OR 中风后运动功能障碍 OR 生活质量 OR 平衡功能 OR 功能障碍 OR 运动功能 OR 卒中 OR 脑卒中 OR 中风) and 主题:(太极 OR 太极拳 OR 八段锦 OR 五禽戏 OR 易筋经 OR 气功 OR 健身气功) and 摘要:(FMA OR BBS OR BI)
