# Supplementary material for: Comparison of the effects of different traditional Chinese exercises on improving the motor function of stroke survivors: a network meta-analysis and systematic review
Source: Front Neurol. 2026 Jun 24;17:1815489. doi: 10.3389/fneur.2026.1815489 (PMC13341441; doi:10.3389/fneur.2026.1815489)
Supplement: Supplementary file 2 [file Supplementary_file_2.PDF]

Appendix b Inclusion of 50 studies

REFERENCE：

(1-50)

1. JI Xiaoyu; YUAN Meng; SONG Yan. Effects of Baduanjin intervention on motor function, electromyography characteristics and serum neural cytokine levels in stroke patients with hemiplegia. Clinical Research and Practice 2022, 7(20)18-22. DOI:10.19347/j.cnki.2096-1413.202220005.

2. LING Yan; MAO Zhenzhu. Baduanjin on motor function of mild acute ischemic stroke and the impact of quality of life. Research of Integrated Traditional Chinese and Western Medicine 2024, 16(6)428-430. DOI:10.3969/j.issn.1674-4616.2024.06.015.

3. DU Tengfei; LI Xin; WU Xiaowei. Effect of Baduanjin on Postural Control and Balance i n Stroke Patients During the Recovery Phase. New Chinese Medicine 2025, 57(15)56-61. DOI:10.13457/j.cnki.jncm.2025.15.010.

4. HUANG Wenjie; WANG Lijuan; ZHANG Zhengyi; ZHOU Bin. Effect of Baduanjin on limb function,life index and quality of life in convalescent patients with stroke. China Modern Medicine 2025, 18(32)49-52 Available online at: https://qikan.cqvip.com/Qikan/Article/Detail?id=7201377605&from=Qikan_Search_Index

5. XIE Beijing ;YANG Ming; BAI Yulong. Clinical study on the effect of Baduanjin on moto r rehabilitation of stroke patients. West China Medical Journal 2019, 5(34)515-519. Available online at: https://qikan.cqvip.com/Qikan/Article/Detail?id=7002100601&from=Qikan_Search_Index

6. DING Yi; GUO Chenchen; WANG Ning. et al. Effect of Baduanjin’s Third Part Combine d with Conventional Balance Training on Balance Function of Patients with Cerebral Strok. Shandong Journal of Traditional Chinese Medicine. 2019,38(07):673-676+704.DOI:10.16295/j.cnki.0257-358x.2019.07.013.

7. ZHANG Lingling, HUANG Caixia. Effects of Baduanjin rehabilitation training on limb mot or function, daily life and quality of life in elderly stroke patients with hemiplegia. Chine se Journal of Gerontology 2021, 21(41)4620-4622. doi: 10. 3969 /j. issn. 1005-9202. 2021. 21. 006

8. LIU Wan, ZHAO Yan, YANG Dan, FU Qingrong, ZHOU Jing. Effect of Baduanjin exercise prescription on self-dynamic balance of stroke patients in convalescent stage. 2022, 33(8) 1936-1939. Available online at: https://qikan.cqvip.com/Qikan/Article/Detail?id=7108254040&from=Qikan_Search_Index

9. Zhou Haiying; Wu Yunying; Wu Chunlan. Effects of eight trigrams boxing combined wit h rehabilitation training in the treatment of elderly patients with convalescent cerebral va scular accident. Geriatrics & Health Care 2021, 27(6)1191-1194 Available online at: https://qikan.cqvip.com/Qikan/Article/Detail?id=7106304891&from=Qikan_Search_Index

10. WANG Yizhi, WEN Qingxiu, LI Xiuxia. Effect of Baduanjin exercise prescription on bala nce function of stroke patients in convalescent stage. Shenzhen Journal of Integrated Tra ditional Chinese and Western Medicine 2023 22(33)131-133. DOI：10.16458/j.cnki.1007-0893.2023.22.039

11. GUAN Feng. Application effect of baduanj in in elderly patients with stroke in conval

escent stage. China's Naturopathy 2023, 31(24)43-46. DOI:10.19621/j.cnki.11-3555/r.2023.2413.
12. CHEN Jun, ZHANGRuoyi, DINGLingling. Observation on the clinical effect of fixed Taiji exercise in the rehabilitation of 92 patients with stroke hemiplegia. 2022,35(06):1059-1061.DOI:10.19381/j.issn.1001-7585.2022.06.072.
13. Zhao Weiwei; Yao Wang; Deng Guogang; Che Pei; Wu Ming; Huang Guilan; Wang Tong. Impact of modified Baduanjin exercise on lower limb motor control abilities in stroke patients. Chinese Journal of Rehabilitation, 2025,40(07):387-392. DOI: 10.3780/zgkf.2025.07.001.
14.CHEN Junwen; CHEN Qian; CHEN Cheng; LI Shuyue; LIU Lingling; WU Cunshu; GONG Xiang; LU Jun; XU Guangxu. Effect of modified Baduanjin exercise on cardiopulmonary function,motor function and activities of daily living for stroke patients. Chinese Journal of Rehabilitation Theory and Practice. 2024,30(01):74-80. DOI: 10.3969/j.issn.1006-9771.2024.01.010
15. FAN Jing; GUO Yue-Ping; GUO Peng-Fei. Effect of modified Tai Chi on balance in stroke patients and on serum alkaline phosphatase, neuropeptide Y and interleukin-6 expression. Chinese Journal of Multiple Organ Diseases in the Elderly. 2020,19(02):103-108. DOI: 10.11915/j.Issn.1671-5403.2020.02.026
16. Zhou Li; Li Zongheng; Zhang Yong; Chen Kai; Lin Yin. Preliminary Study on the Effect of Modified Tai Chi for Motor Function in Patients with Stroke. Chinese Journal of Integrative Medicine on Cardio-Cerebrovascular Disease. 2015,13(07):878-880. DOI：10.3969/.issn.1672-1449.2025.07.005
17. ZHANG Chaolong, YANG Jianfei, ZHANG Hefei. Effect of modified Wuqinxi on the degree of nerve defect and balance ability in elderly patients with stroke, 2025,38(05):37-41. DOI: 10.3969/j.issn.1001-6910.2025.05.1
18. CHE Pei, BAO Yingying, JI Yingying. Precise assessment and analysis of the effects of the modified Wuqinxi on balance function in stroke patients. Chinese Journal of Rehabilitation Medicine, 2024,39(06):828-834. DOI:10.3969/j.issn.1001-1242.2024.06.010
19. ZHANG Fenglin; SHU Hongyan; REN Leilei; et,al. Study on efficacy of modified Wuqinxi on gait and balance dysfunction in elderly stroke patients and on brain mechanism based on fMRI. CHEN Xianbing.The Affiliated Hospital of Zunyi Medical Uinversity. 2023,39(09):1174-1178. DOIi：10.3969/j.issn.1006-5725.2023.09.020.
20. HOU Cuilan; ZHONG Weihong; YANG Jingda. et,al. Correlation Study of Baduanjin on Core and Balance Function in Stroke Patients Based on Surface EMG. Traditional Chinese Medicine Rehabilitation. 2025,2(05):7-12.DOI:10.19787/j.issn.2097-3128.2025.05.002.
21. CUI Yongsheng，WANG Meijuan YANG Huixin. Effect of health Qigong Baduanjin on motor function of stroke patients during recovery period. Journal of Shandong Sport University. 2018,34(03):97-100.DOI:10.14104/j.cnki.1006-2076.2018.03.015.
22. ZHANG Jing, JIA Weizong, LI Haiying. et,al. Study on the effect of health qigong Yijinjing exercise on improving the motor function of stroke patients with hemiplegia. Sport. 2010,(12):123-124+86. DOI:10.3969/j.issn.1674-151x.2010.12.065.
23. HE Jing; WANG Wu; LI Kunpeng. et,al. The effect of six-forms Tai Chi training on the postural control of stroke survivors. Chinese Journal of Rehabilitation Medicine. 2022,37(04):482-487. DOI:10.3969/j.issn.1001-1242.2022.04.008

24. YANG Zhibo, LIU Dong, CHANG Yuesong. Clinical Study on the Treatment of Hemiplegic Balance Disorder after Stroke by Tai Chi Balance Method. Contemporary Medicine, 2013,19(24):5-7. DOI: 10.3969/j.issn.1009-4393.2013.24.003.

25. FU Changxi, ZHANG Qiuyang. Effects of taijiquan on balance function and walking ability of stroke hemiplegic patients in convalescent phase. Chinese Journal of Rehabilitation Medicine. 2016,31(05):536-539. DOI:10.3969/j.issn.1001-1242.2016.05.008

26. LIU Jia. Effect of Tai Chi on limb function and balance function in patients with ischemic stroke. Capital Food Medicine. 2019,26(23):132. Available online at: https://qikan.cqvip.com/Qikan/Article/Detail?id=7100513383&from=Qikan_Search_Index

27. ZHAO Bin, TANG Qiang, WANG Yan. Effects of Taijiquan on Motor Function and Depression in Patients with Post-stroke Depression. Chinese Journal of Rehabilitation Theory and Practice. 2017,23(03):334-337. DOI: 10.3969/j.issn.1006-9771.2017.03.019.

28. ZHANG Jiyun; LI Lin. Effect of Tai Chi Rehabilitation Training on Posture Balance Function of Stroke Patients. Smart Healthcare. 2024,10(30):175-178.DOI:10.19335/j.cnki.2096-1219.2024.30.051.

29. WANG Xinyuan. Effect of Taiji Cloud Hand Training on upper Limb Dysfunction after Stroke. Clinical Research. 2021,29(06):106-108. Available online at: https://qikan.cqvip.com/Qikan/Article/Detail?id=7104637978&from=Qikan_Search_Index

30. JIANG Suzhen CHEN Jinxiu LU Weini. The effects of Tai Chi Yunshou exercises on upper extremity function in stroke patients with hemiplegia. Chinese Journal of Nursing Education. 2018,15(03):219-222. DOI：10.3761/j.issn.1672-9234.2018.03.015

31. XU Xiaodong, BAIJing, ZHANG Hongli, et al. Analysis of curative effect for Tai chi on the balance function of patients with hemiplegia after stroke. Hebei Journal of Traditional Chinese Medicine. 2014,36(08):1149-1150+1198. Available online at: https://qikan.cqvip.com/Qikan/Article/Detail?id=661872437&from=Qikan_Search_Index

32. LAI Jiangong, LAI Meijin, LI Baofan. et al. To explore the clinical study on the effect of Chen-style Taijiquan specific movements on lower limb walking dysfunction in stroke patients. Contemporary Medical Symposium 2024,22(25):126-129 Available online at: https://qikan.cqvip.com/Qikan/Article/Detail?id=7113029120&from=Qikan_Search_Index

33. WANG Wuhao; ZHANG Guangpeng; XIE Haijiang. et al. Effect of Sitting Tai Chi on Upper Limb Motor Function in Brunnstrom Ⅱ Stroke Patients. Journal of Chengdu Sport University. 2023,49(02):82-87.DOI:10.15942/j.jcsu.2023.02.012.

34. Xie Q, Wu J, Zhang Q, et al. Neurobiomechanical mechanism of Tai Chi to improve upper limb coordination function in post-stroke patients: a study protocol for a randomized controlled trial. Trials. 2023;24(1):788. Published 2023 Dec 4. doi:10.1186/s13063-023-07743-w.

35. YANG Huixin, TANG Qiang. Clinical Observation of Tai Chi for Rehabilitation of Motor Dysfunction in Stroke Patients. Chinese Journal of Rehabilitation Medicine 2016,31(10):1146-1148. DOI:10.3969/j.issn.1001-1242.2016.10.021

36. YANG Huixin;LIU Xiaolei. Effects of Taiji Quan and Baduanjin on Motor Function of Lower Limbs for Stroke Patients using Surface Electromyography. Chinese Journal of Rehabilitation Theory and Practice, 2019,25(1):101-106 DOI: 10.3969/j.issn.1006- 9771.2019.01.014

37. LI Yang; WANG Chun; LIU Chao. et al. Effect of Tai Chi Lower Limb Movement Exercises on Balance Function in Patients with Brunnstrom Stage IV Stroke. Fujian Sports Scien

ce and Technology, 2025,44(4):59-6573 Available online at: https://qikan.cqvip.com/Qikan/Article/Detail?id=7201864480&from=Qikan_Search_Index
38. ZHENG Haiying; ZHOU Hao; YANG Dianlong. Clinical Observation of Tai Chi Training Combined with Routine Rehabilitation Training in the Treatment of Post-stroke Balance Dysfunction Journal of Liaoning University of Traditional Chinese Medicine 2020,22(9):19-22 DOI:10.13194/j.issn.1673-842x.2020.09.006.
39. ZHU Junrong; LI Shuying; WANG Ning. et al. Effects of Short-term Baduanjin Exercise and Core Stability Training on Balance Function in Convalescent Stroke Patients Chinese Journal of Integrative Medicine on Cardio-Cerebrovascular Disease 2025,23(2):300-304 DOI：10.12102/j.issn.1672-1349.2025.02.026
40. LIU Wenli, HE Aihua, WU Wenting. et al. Effect analysis of modified sitting Baduanjin combined with exercise rehabilitation in the treatment of elderly stroke patients with hemiplegia. Modern Medicine and Health Research, 2025,9(7):28-30 DOI：10.3969/j.issn.2096-3718.2025.07.009
41. LUO Kailiang; JIN Xueming; MA Shujie. et al. Effect of Yijinjing Training on Balance Function and Plantar Pressure of Stroke Patients. Rehabilitation Medicine. 2022,32(2):117-123 DOI：10.3724/SP.J.1329.2022.02005
42. XU Jin; ZHANG Yu; XU Wen-xiu. Effect of Early Rehabilitation with YiJinJing Exercise on Barthel Index,FMA and SF-36 in Patients with Severe Hemiplegic Stroke. Lingnan Journal of Emergency Medicine. 2022,27(6):521-523 DOI：10.3969/j.issn.1671-301X.2022.06.007
43. XU Lin;bHU Ju. The Effect of Modified Yijinjing Adjuvant Therapy on Balance Ability and Fall Efficacy in Stroke Patients. Reflexology And Rehabilitation Medicine. 2023,4(18):76-78 Available online at: https://qikan.cqvip.com/Qikan/Article/Detail?id=00004H0G596NJEKK5D3F1JH1MLD08IP1MLD9Q&from=Qikan_Search_Index
44. TANG Linxia; GONG Bomin; SHI Zhenwen; WANG Bin. Effect of qigong practice wuqinxi on the motor function of elderly patients with stroke hemiplegia. China Medical Herald. 2018,15(25):112-115 Available online at: https://qikan.cqvip.com/Qikan/Article/Detail?id=676233751&from=Qikan_Search_Index
45. Chen Shi; Zhou Yang; Sun Yuwei; Quan Lu; Xi Hang. Study on the Effect of Improving the Yijin Jing on the Rehabilitation of Upper Limb Function in Stroke Patients during the Recovery Period. Technology and Health. 2024,3(22):59-62 Available online at: https://qikan.cqvip.com/Qikan/Article/Detail?id=7113612612&from=Qikan_Search_Index
46. ZHANG Xiaofan; ZHANG Wenfang; YAN Chenjing; NING Shuping. Analysis of the Application Effect of Improved Yijinjing Therapy in Upper Limb Rehabilitation of Stroke Patients. Chinese Journal of Trauma and Disability Medicine. 2025,33(5):26-30 Available online at: https://qikan.cqvip.com/Qikan/Article/Detail?id=7200596491&from=Qikan_Search_Index
47. WEN Pengzhen; LIU Jun; LIAO Wenzhou. Observation on the therapeutic effect of Huatuo Wuqinxi training camp on post-stroke depression patients. Proceeding of Clinical Medicine. 2024,33(9):661-664 DOI:10.16047/j.cnki.cn14-1300/r.2024.09.004.
48. CHEN Qin; ZHANG Xiaofei; ZHANG Qilin. Clinical Effects of Taiji Massage Combined with Electromyographic Biofeedback Therapy on Upper Limb Motor Dysfunction after Stroke. Medical Innovation of China. 2025,22(5):43-47 DOI: 10.3969/j.issn.1674-4985.2025.05.010
49. ZHANG Shu, ZONG Xinxin. Study on the effect of Baduanjin intervention on the quality of life of patients with stroke recovery period.Modern Medicine and Health Research.

2025,9(24):96-99 DOI: 10.3969/j.issn.2096-3718.2025.24.029
50. CHEN Jun, LIN Leilei, ZHONG Haibo, et al. Observation on the curative effect of modified Wuqinxi combined with graded motor imagery training in the treatment of upper limb hemiplegia after stroke. Zhejiang Journal of Traditional Chinese Medicine. 2025,60(11): 995-996 DOI: 10.13633/j.cnki.zjtcm.2025.11.024
