# Supplementary material for: Comparison of the effects of different traditional Chinese exercises on improving the motor function of stroke survivors: a network meta-analysis and systematic review
Source: Front Neurol. 2026 Jun 24;17:1815489. doi: 10.3389/fneur.2026.1815489 (PMC13341441; doi:10.3389/fneur.2026.1815489)
Supplement: Supplementary file 3 [file Supplementary_file_3.PDF]

Table a1 FMA-UE League Table

MD 95%CI

BDJ				
21.68 (0.18, 2438.62)	SOC			
0 (0, 12.2)	0 (0, 0.14)	TC		
0 (0, 61.81)	0 (0, 0.88)	1.86 (0, 168752.33)	WQX	
0.01 (0, 87.67)	0 (0, 1.22)	2.85 (0, 241436.54)	1.54 (0, 329516.71)	YJJ

Table a2 FMA-LE League Table

MD 95%CI

BDJ				
15.28 (1.42, 170.19)	SOC			
0.71 (0.02, 26.12)	0.05 (0, 0.67)	TC		
0.08(0, 14)	0.01 (0, 0.51)	0.11(0, 22.65)	WQX	
4.74 (0.01, 3992.03)	0.31 (0, 168.19)	6.59 (0.01,6371.65)	59.21 (0.02, 127538.35)	YJJ

Table a3 BBS League Table

MD 95%CI

BDJ				
78.81 (12.17, 528.4)	SOC			
0.4 (0.03, 6.31)	0.01 (0, 0.04)	TC		
0.67 (0.01, 33.25)	0.01 (0, 0.25)	1.69 (0.03, 87.64)	WQX	
0.23 (0, 40.02)	0 (0, 0.35)	0.58 (0, 104.89)	1.54 (0, 329516.71)	YJJ

Table a4 BI League Table

MD 95%CI

BDJ				
333597.91 (2232.09, 36655842.46)	SOC			
332.21 (0.32, 255732.72)	0 (0, 0.11)	TC		
68.5 (0.01, 602114.75)	0 (0, 0.53)	0.2 (0, 2021.02)	WQX	
1.02 (0, 2964.38)	0 (0, 0)	0 (0, 9.42)	0.02 (0, 420.1)	YJJ

$p<0.05$ signifies statistical significance (marked in light blue)
