# Supplementary material for: Comparison of the effects of different traditional Chinese exercises on improving the motor function of stroke survivors: a network meta-analysis and systematic review
Source: Front Neurol. 2026 Jun 24;17:1815489. doi: 10.3389/fneur.2026.1815489 (PMC13341441; doi:10.3389/fneur.2026.1815489)

Appendix d Pairwise comparison

BBS pairwise comparison

Comparison	Number of studies	Heterogeneity	MD (95%CI)
**SOC vs BDJ**	13	95.6%	-4.3671 (-6.2698, -2.4927)
**SOC vs TC**	12	98.6%	5.2859 (3.2877, 7.3240)
**SOC vs WQX**	4	54.6%	4.7565 (1.3696, 8.1521)
**SOC vs YJJ**	2	0.0%	5.8197 (1.0167, 10.593)

BI pairwise comparison

Comparison	Number of studies	Heterogeneity	MD (95%CI)
**SOC vs BDJ**	6	90.4%	-12.709 (-17.439, -7.6291)
**SOC vs TC**	6	69.7%	6.9232 (2.1907, 11.655)
**SOC vs WQX**	2	39.1%	8.4960 (0.61114, 16.512)
**SOC vs YJJ**	3	91.8%	12.679 (6.1486, 19.508)

FMA-UE pairwise comparison

Comparison	Number of studies	Heterogeneity	MD (95%CI)
**SOC vs BDJ**	9	97.8%	-3.0837 (-7.7719, 1.6478)
**SOC vs TC**	4	99.9%	9.4106 (1.9875, 16.726)
**SOC vs WQX**	3	62.2%	3.3923 (0.65755, 6.1721)
**SOC vs YJJ**	3	92.8%	8.3654 (−0.17282, 16.865)

FMA-LE pairwise comparison

Comparison	Number of studies	Heterogeneity	MD (95%CI)
**SOC vs BDJ**	7	95.6%	-2.7319 (-5.1445, -0.3247)
**SOC vs TC**	6	91.9%	3.0590

			(0.37077, 5.7396)
**SOC vs WQX**	2	84.2%	5.2507 (0.67723, 9.7519)
**SOC vs YJJ**	1	-	1.1776 (-5.1438, 7.4458)

Figure a1: Funnel Plots of BBS

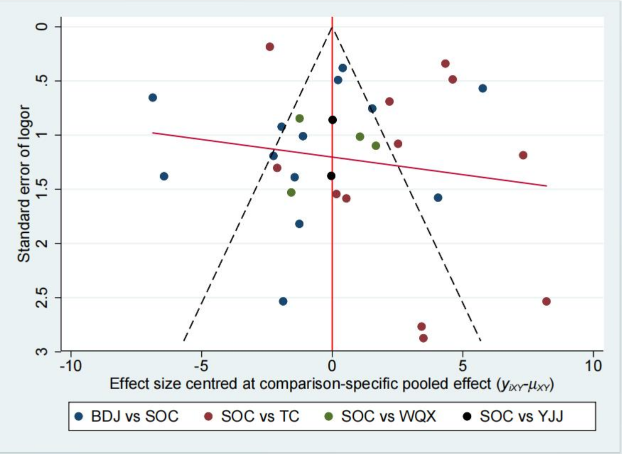

Figure a2: Funnel Plots of BI

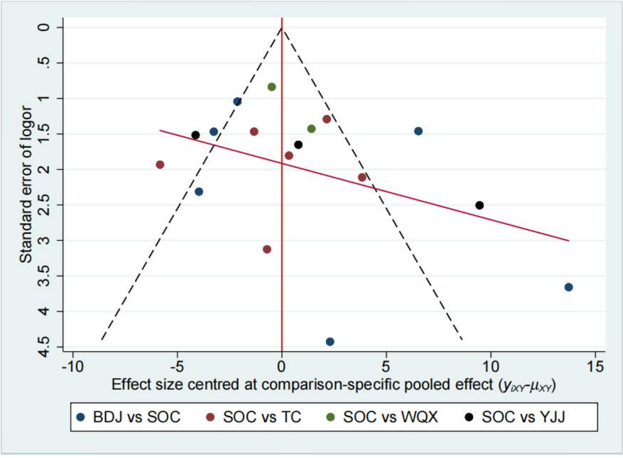

Figure a3: Funnel Plots of FMA-UE

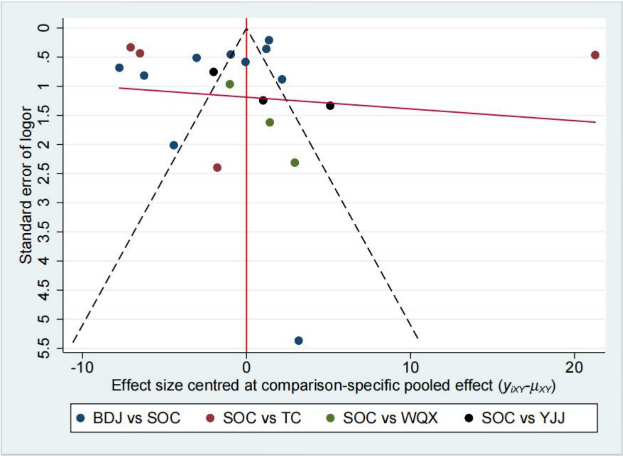

Figure a4: Funnel Plots of FMA-LE

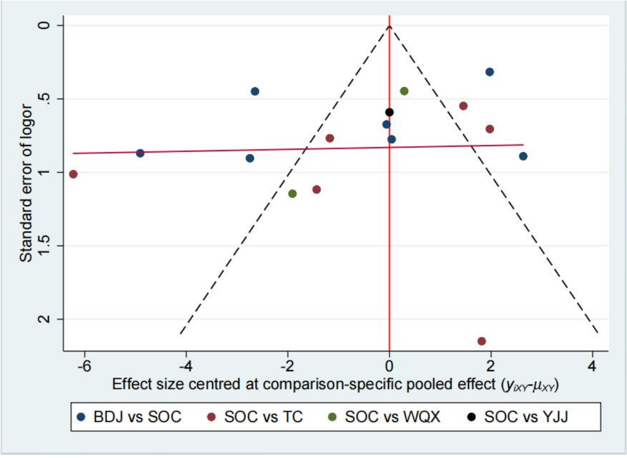

Supplement: Supplementary file 4 [file Supplementary_file_4.PDF]
