# Supplementary material for: Comparison of the effects of different traditional Chinese exercises on improving the motor function of stroke survivors: a network meta-analysis and systematic review
Source: Front Neurol. 2026 Jun 24;17:1815489. doi: 10.3389/fneur.2026.1815489 (PMC13341441; doi:10.3389/fneur.2026.1815489)
Supplement: Supplementary file 5 [file Supplementary_file_5.pdf]

Summary of the risk of bias

Study	***Generation of random sequences***	***Allocation concealment***	***Blinding***	***Blinding of outcome evaluators***	***Incomplate data***	***Selective repetoring***	***Other bias***
Ji 2022	*low risk*	*unclear risk*	*unclear risk*	*unclear risk*	*low risk*	*low risk*	*unclear risk*
Ling 2024	*low risk*	*unclear risk*	*unclear risk*	*unclear risk*	*high risk*	*low risk*	*unclear risk*
Du 2025	*low risk*	*unclear risk*	*unclear risk*	*unclear risk*	*low risk*	*low risk*	*unclear risk*
Huang 2025	*low risk*	*unclear risk*	*unclear risk*	*unclear risk*	*low risk*	*low risk*	*unclear risk*
Xie 2019	*low risk*	*unclear risk*	*low risk*	*low risk*	*low risk*	*low risk*	*unclear risk*
Ding 2019	*low risk*	*unclear risk*	*low risk*	*unclear risk*	*high risk*	*low risk*	*unclear risk*
Zhang 2021	*low risk*	*unclear risk*	*unclear risk*	*unclear risk*	*low risk*	*low risk*	*unclear risk*
Liu 2022	*low risk*	*unclear risk*	*unclear risk*	*unclear risk*	*low risk*	*low risk*	*unclear risk*
Zhou 2021	*low risk*	*unclear risk*	*unclear risk*	*unclear risk*	*low risk*	*low risk*	*unclear risk*
Wang 2023	*low risk*	*low risk*	*unclear risk*	*unclear risk*	*high risk*	*low risk*	*unclear risk*
Guan 2023	*low risk*	*unclear risk*	*unclear risk*	*unclear risk*	*low risk*	*low risk*	*unclear risk*
Chen 2022	*low risk*	*unclear risk*	*unclear risk*	*unclear risk*	*low risk*	*low risk*	*unclear risk*
Zhao 2025	*unclear risk*	*unclear risk*	*unclear risk*	*unclear risk*	*unclear risk*	*low risk*	*unclear risk*
Chen 2024	*low risk*	*unclear risk*	*unclear risk*	*unclear risk*	*low risk*	*low risk*	*unclear risk*
Fan 2020	*low risk*	*unclear risk*	*unclear risk*	*unclear risk*	*low risk*	*low risk*	*unclear risk*
Zhou 2015	*low risk*	*unclear risk*	*unclear risk*	*unclear risk*	*low risk*	*low risk*	*unclear risk*
Zhang 2025	*unclear risk*	*unclear risk*	*unclear risk*	*unclear risk*	*low risk*	*low risk*	*unclear risk*
Che 2024	*low risk*	*unclear risk*	*unclear risk*	*unclear risk*	*unclear risk*	*low risk*	*unclear risk*
Zhang 2023	*low risk*	*unclear risk*	*unclear risk*	*unclear risk*	*low risk*	*low risk*	*unclear risk*
Hou 2025	*low risk*	*unclear risk*	*low risk*	*low risk*	*low risk*	*low risk*	*unclear risk*
Cui 2018	*unclear risk*	*unclear risk*	*unclear risk*	*unclear risk*	*high risk*	*low risk*	*unclear risk*
Zhang 2010	*unclear risk*	*unclear risk*	*unclear risk*	*unclear risk*	*low risk*	*low risk*	*unclear risk*
He 2022	*low risk*	*unclear risk*	*unclear risk*	*unclear risk*	*high risk*	*low risk*	*unclear risk*
Yang 2013	*low risk*	*unclear risk*	*unclear risk*	*unclear risk*	*low risk*	*low risk*	*unclear risk*
Fu 2016	*unclear risk*	*unclear risk*	*unclear risk*	*unclear risk*	*low risk*	*low risk*	*unclear risk*
Liu 2019	*unclear risk*	*unclear risk*	*unclear risk*	*unclear risk*	*unclear risk*	*unclear risk*	*unclear risk*
Zhao 2017	*low risk*	*unclear risk*	*unclear risk*	*unclear risk*	*low risk*	*low risk*	*unclear risk*
Zhang 2024	*low risk*	*unclear risk*	*unclear risk*	*unclear risk*	*low risk*	*low risk*	*unclear risk*
Wang 2021	*low risk*	*unclear risk*	*unclear risk*	*unclear risk*	*high risk*	*low risk*	*unclear risk*
Jiang 2018	*high risk*	*unclear risk*	*unclear risk*	*unclear risk*	*low risk*	*low risk*	*unclear risk*
Xu 2014	*unclear risk*	*unclear risk*	*unclear risk*	*unclear risk*	*low risk*	*low risk*	*unclear risk*

Lai 2024	*unclear risk*	*unclear risk*	*unclear risk*	*unclear risk*	*high risk*	*high risk*	*unclear risk*
Wang 2023	*unclear risk*	*unclear risk*	*unclear risk*	*unclear risk*	*high risk*	*low risk*	*unclear risk*
Xie 2023	*low risk*	*unclear risk*	*low risk*	*unclear risk*	*low risk*	*low risk*	*unclear risk*
Yang 2016	*unclear risk*	*unclear risk*	*unclear risk*	*unclear risk*	*high risk*	*low risk*	*unclear risk*
Yang 2019	*unclear risk*	*unclear risk*	*unclear risk*	*unclear risk*	*high risk*	*low risk*	*unclear risk*
Li 2025	*low risk*	*unclear risk*	*unclear risk*	*unclear risk*	*low risk*	*low risk*	*unclear risk*
Zheng 2020	*low risk*	*unclear risk*	*unclear risk*	*unclear risk*	*low risk*	*low risk*	*unclear risk*
Zhu 2025	*low risk*	*unclear risk*	*unclear risk*	*unclear risk*	*low risk*	*low risk*	*unclear risk*
Liu 2025	*unclear risk*	*unclear risk*	*unclear risk*	*unclear risk*	*low risk*	*low risk*	*unclear risk*
Luo 2022	*low risk*	*low risk*	*low risk*	*unclear risk*	*low risk*	*low risk*	*unclear risk*
Xu 2022	*unclear risk*	*unclear risk*	*unclear risk*	*unclear risk*	*low risk*	*low risk*	*unclear risk*
Xu 2023	*unclear risk*	*unclear risk*	*unclear risk*	*unclear risk*	*low risk*	*low risk*	*unclear risk*
Tang 2018	*low risk*	*unclear risk*	*unclear risk*	*unclear risk*	*low risk*	*low risk*	*unclear risk*
Chen 2024	*low risk*	*unclear risk*	*unclear risk*	*unclear risk*	*low risk*	*low risk*	*unclear risk*
Zhang 2025	*low risk*	*unclear risk*	*unclear risk*	*unclear risk*	*low risk*	*low risk*	*unclear risk*
Wen 2024	*low risk*	*unclear risk*	*unclear risk*	*unclear risk*	*low risk*	*low risk*	*unclear risk*
Chen 2025	*low risk*	*unclear risk*	*unclear risk*	*unclear risk*	*low risk*	*low risk*	*unclear risk*
Zhang 2025	*low risk*	*unclear risk*	*unclear risk*	*unclear risk*	*low risk*	*low risk*	*unclear risk*
Chen 2025	*low risk*	*unclear risk*	*unclear risk*	*unclear risk*	*high risk*	*low risk*	*unclear risk*
